# Transcriptome Analysis of *Nicotiana tabacum* Infected by *Cucumber mosaic virus* during Systemic Symptom Development

**DOI:** 10.1371/journal.pone.0043447

**Published:** 2012-08-28

**Authors:** Jie Lu, Zhi-Xin Du, Jun Kong, Ling-Na Chen, Yan-Hong Qiu, Gui-Fen Li, Xiao-Hua Meng, Shui-Fang Zhu

**Affiliations:** 1 Institute of Plant Quarantine, Chinese Academy of Inspection and Quarantine, Beijing, China; 2 College of Agronomy and Biotechnology, China Agricultural University, Beijing, China; 3 Beijing Genomics Institute-Shenzhen, Shenzhen, China; Max Planck Institute for Chemical Ecology, Germany

## Abstract

Virus infection of plants may induce a variety of disease symptoms. However, little is known about the molecular mechanism of systemic symptom development in infected plants. Here we performed the first next-generation sequencing study to identify gene expression changes associated with disease development in tobacco plants (*Nicotiana tabacum* cv. Xanthi nc) induced by infection with the M strain of *Cucumber mosaic virus* (M-CMV). Analysis of the tobacco transcriptome by RNA-Seq identified 95,916 unigenes, 34,408 of which were new transcripts by database searches. Deep sequencing was subsequently used to compare the digital gene expression (DGE) profiles of the healthy plants with the infected plants at six sequential disease development stages, including vein clearing, mosaic, severe chlorosis, partial and complete recovery, and secondary mosaic. Thousands of differentially expressed genes were identified, and KEGG pathway analysis of these genes suggested that many biological processes, such as photosynthesis, pigment metabolism and plant-pathogen interaction, were involved in systemic symptom development. Our systematic analysis provides comprehensive transcriptomic information regarding systemic symptom development in virus-infected plants. This information will help further our understanding of the detailed mechanisms of plant responses to viral infection.

## Introduction

Understanding the responses of plant hosts to viral infection is important for developing strategies for disease control. Since plant responses are complex and relate to a large variety of physiological processes, system-level transcriptomic studies are powerful for fully understanding plant responses [1], [2]. One of the most established techniques for studying the transcriptome, gene expression microarrays, has been used to reveal gene expression profiles in *Arabidopsis thaliana* after infection with plant viruses [3]–[7]. Responses to virus infection have also been monitored in other host plant species with expression microarrays [8]–[16]. While these studies have revealed gene expression changes in response to virus infection, they are less informative in providing system-level transcriptional responses due to the technical limitations of microarrays.

Recently, next-generation deep-sequencing techniques such as Solexa/Illumina RNA-Seq and digital gene expression (DGE) have provided new approaches for studying the transcriptome, and have several advantages over microarray analysis [17]–[20]. RNA-Seq is a whole transcriptome sequencing method that sequences the overlapping short fragments obtained from mRNA or cDNA to give a quantitative description of the entire transcriptome. RNA-Seq can measure gene expression at the transcriptional level, identify genes, non-coding and new transcription units, and determine the structure of transcripts, thus providing large volumes of new information on the complexity and dynamics of transcriptomes [21]–[24]. DGE sequences short tags (usually about 20 bp) generated by endonucleases from the 3′ ends of genes and the copy number of each tag indicates the expression level of the corresponding gene. This tag-based sequencing method is particularly suitable and cost-effective for genome-wide expression profiling to analyze gene expression levels [25]–[27]. To date, many transcriptome studies have been carried out using RNA-Seq and/or DGE and these studies have greatly extended our knowledge of the complexity of eukaryotic transcriptomes [21]–[30].

*Cucumber mosaic virus* (CMV) is one of the most important plant pathogens, can infect more than 1,200 plant species [31], [32], and is frequently used in genetic studies to investigate host resistance responses in *A. thaliana* [4], [5], [33]. There are many strains of CMV, and different strains can induce different symptoms in *Nicotiana tabacum*. M strain of *Cucumber mosaic virus* (M-CMV) is highly virulent to the tobacco plants, but the disease development process includes a transient recovery period when the newly emerged leaves appear nearly healthy. Thus, M-CMV infection of Xanthi tobacco plants provides an ideal model for investigating host gene expression changes associated with disease induction. The study on M-CMV-infected Xanthi tobaccos will provide clues to answer some important biological questions related to the symptom development processes of plant disease: which are the predominant biological processes in the initial pathogenesis, transient recovery and secondary pathogenesis processes? What are the differences between transient recovery process and previously reported plant recovery induced by antiviral RNA silencing?

In the present study, we analyzed responses of Xanthi tobacco plants (*Nicotiana tabacum* cv. Xanthi nc) to infection by M-CMV at the transcriptome level using next-generation deep sequencing approaches. We investigated the gene expression changes between virus-infected samples and mock-inoculated samples at different symptom development stages after M-CMV infection, including vein clearing, mosaic, severe chlorosis, partial recovery, total recovery and re-mosaic. The results indicated that photosynthesis and pigment metabolism were suppressed during the initial pathogenesis process and secondary pathogenesis process, and the innate immunity process was significantly enhanced during the transient recovery process. Our study provided some insights to reveal the molecular mechanism of system symptom development on tobacco plants, which would further the understanding of plant-virus interactions.

## Results

### Distinct Stages in the Disease Development Induced by M-CMV Infection

**Figure 1:**
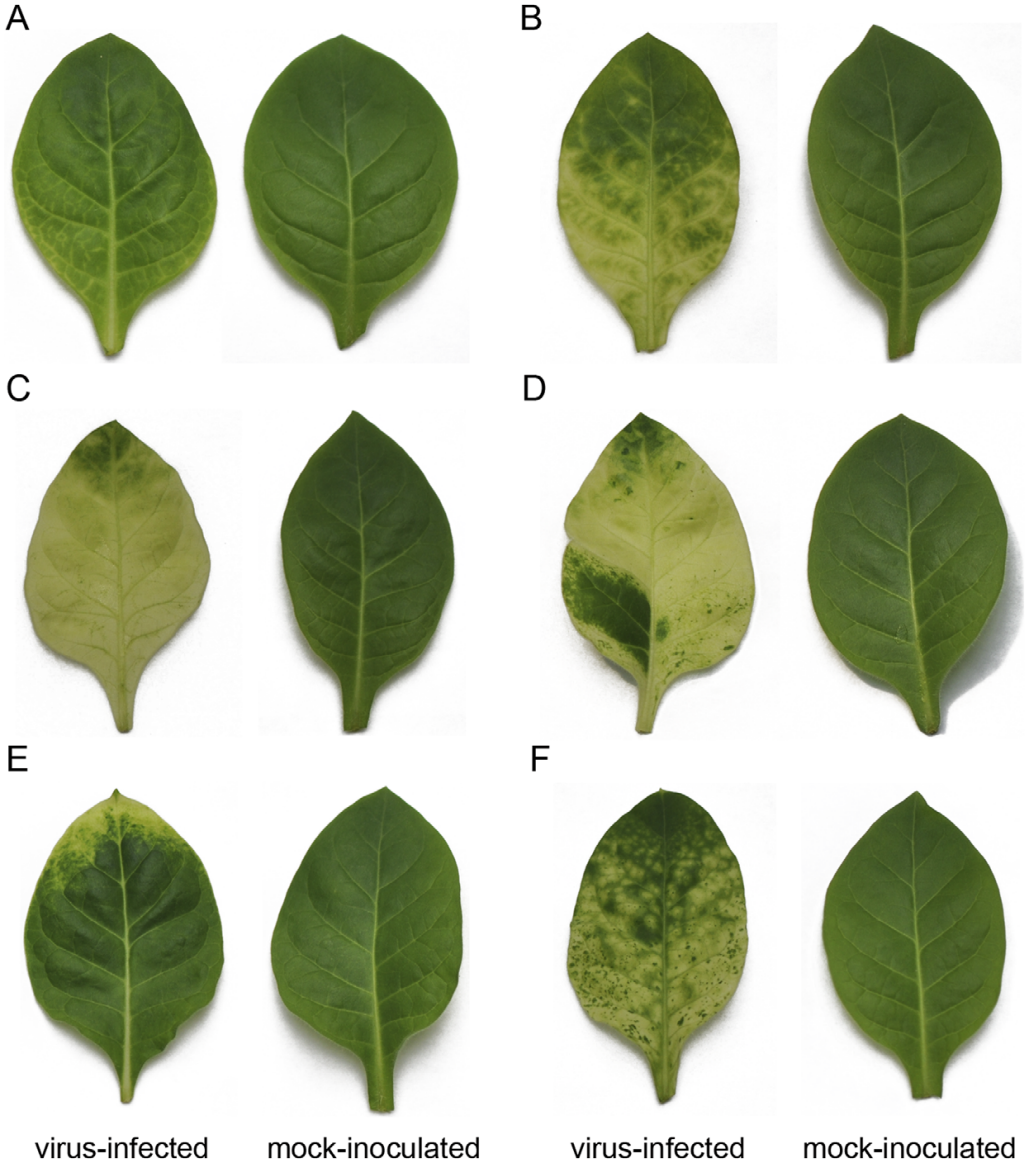
Symptom development in tobacco leaves infected with M-CMV. Virus-infected and mock-inoculated leaves were collected and photographed at 6 dpi (vein clearing, A), 9 dpi (mosaic, B), 11 dpi (severe chlorosis, C), 13 dpi (partial recovery, D), 16 dpi (complete recovery, E) and 20 dpi (secondary mosaic, F).

We chose M-CMV infection of tobacco plants as a model to investigate host transcriptome responses because the disease development can be divided into six visually distinct stages. Under our greenhouse conditions, the leaf immediately above the inoculated leaves initially shows vein clearing 5–7 days post inoculation (dpi, Stage 1, Fig. 1A), which is subsequently developed to the mosaic symptom by 8–10 dpi (Stage 2, Fig. 1B). The next leaf emerged at 11–12 dpi shows severe chlorosis (Stage 3, Fig. 1C), but contains normal green (or partially recovered) regions at 13 dpi (Stage 4, Fig. 1D). Finally, the new leaf emerged at 16 dpi often shows near complete recovery (Stage 5, Fig. 1E), but the next new-emerged leaf will exhibit the typical mosaic symptom again by 18 dpi (Stage 6, Fig. 1F). Thus, the disease development induced by M-CMV includes the initial and secondary pathogenesis processes that are interrupted by a transient recovery. In this study, leaves above the inoculated leaves or emerged after inoculation with M-CMV representing the six stages were collected for RNA extraction on 6, 9, 11, 13, 16 and 20 dpi, respectively. Leaves of a similar size and developmental stage to the collected virus-infected leaves were sampled at the same time from the mock-inoculated tobacco plants and used as controls.

In order to evaluate the viral concentration in leaves representing the above six symptom stages, we performed western blot analysis using monoclonal antibodies of M-CMV particles. As shown in Figure S1, the M-CMV particles were accumulated at significantly high level in leaves at 6, 9, 11 and 13 dpi. There were much fewer M-CMV particles in leaves at 20 dpi compared to that of 6, 9, 11 and 13 dpi. The viral concentration in leaves at 16 dpi was the lowest in the six symptoms.

### Annotation of Unigenes in Tobacco

Since the genome sequence of *N. tabacum* is not available, we first defined the transcriptome by RNA-Seq. To this end, equal quantities of total RNA from the six infected samples and the six samples from the mock-inoculated plants were pooled and used in the library construction for sequencing by Illumina HiSeq 2000. A total of 23,172,654 clean reads (a total of 2,085,538,860 nucleotides) were generated by Illumina RNA-Seq deep-sequencing and assembled into 533,594 contigs, from which 159,069 scaffolds were obtained. After gap filling with paired-end reads, the scaffolds were assembled into 95,916 unigenes. The distribution of unigene size is shown in Figure S2. BLAST results were obtained for a total of 52,529 unigenes against five protein databases, namely the non-redundant (nr, NCBI) protein database, Swiss-Prot database, Kyoto Encyclopedia of Genes and Genomes (KEGG) database and Clusters of Orthologous Groups of proteins (COG, NCBI) database and the Gene Ontology (GO) database (Table 1).

**Table 1:** Summary for BLAST results of unigenes.

Database	Number of unigenes with BLAST results	Percentage of unigenes with BLAST results (%)
Nr	52,282	99.53
Swiss-Prot	30,326	57.73
KEGG Orthology	20,978	39.94
GO	14,629	27.84
COG	11,672	22.22

Sequences with BLAST hits were further analyzed to obtain their COG functions, GO functions and KEGG pathway annotations. COG analysis indicated that 11,672 unigenes had COG annotations and could be grouped into 24 clusters (Figure S3). 14,629 unigenes had GO annotations and were distributed in 41 functional classes including growth, development, cell death, metabolism, and transcription regulation (Figure S4). Unigenes with KEGG annotations were distributed in 125 KEGG pathways. The top three KEGG pathways containing the largest numbers of unigenes were metabolic pathways, plant-pathogen interactions and biosynthesis of plant hormones (Table 2).

**Table 2:** Top 20 enriched KEGG pathways.

Pathway	Unigenes with pathway annotations (20978)	Pathway ID
Metabolic pathways	4,965 (23.67%)	ko01100
Plant-pathogen interactions	1,633 (7.78%)	ko04626
Biosynthesis of plant hormones	1,100 (5.24%)	ko01070
Spliceosome	948 (4.52%)	ko03040
Biosynthesis of phenylpropanoids	858 (4.09%)	ko01061
Starch and sucrose metabolism	705 (3.36%)	ko00500
Biosynthesis of terpenoids and steroids	643 (3.07%)	ko01062
Biosynthesis of alkaloids derived from shikimate pathway	572 (2.73%)	ko01063
Biosynthesis of alkaloids derived from histidine and purine	510 (2.43%)	ko01065
Phenylpropanoid biosynthesis	510 (2.43%)	ko00940
Biosynthesis of alkaloids derived from ornithine, lysine and nicotinic acid	491 (2.34%)	ko01064
Biosynthesis of alkaloids derived from terpenoid and polyketide	456 (2.17%)	ko01066
Ubiquitin-mediated proteolysis	452 (2.15%)	ko04120
Purine metabolism	437 (2.08%)	ko00230
Endocytosis	403 (1.92%)	ko04144
Ribosome	365 (1.74%)	ko03010
Cysteine and methionine metabolism	361 (1.72%)	ko00270
RNA degradation	358 (1.71%)	ko03018
Glycolysis/Gluconeogenesis	348 (1.66%)	ko00010
Pyrimidine metabolism	348 (1.66%)	ko00240

**Figure 2:**
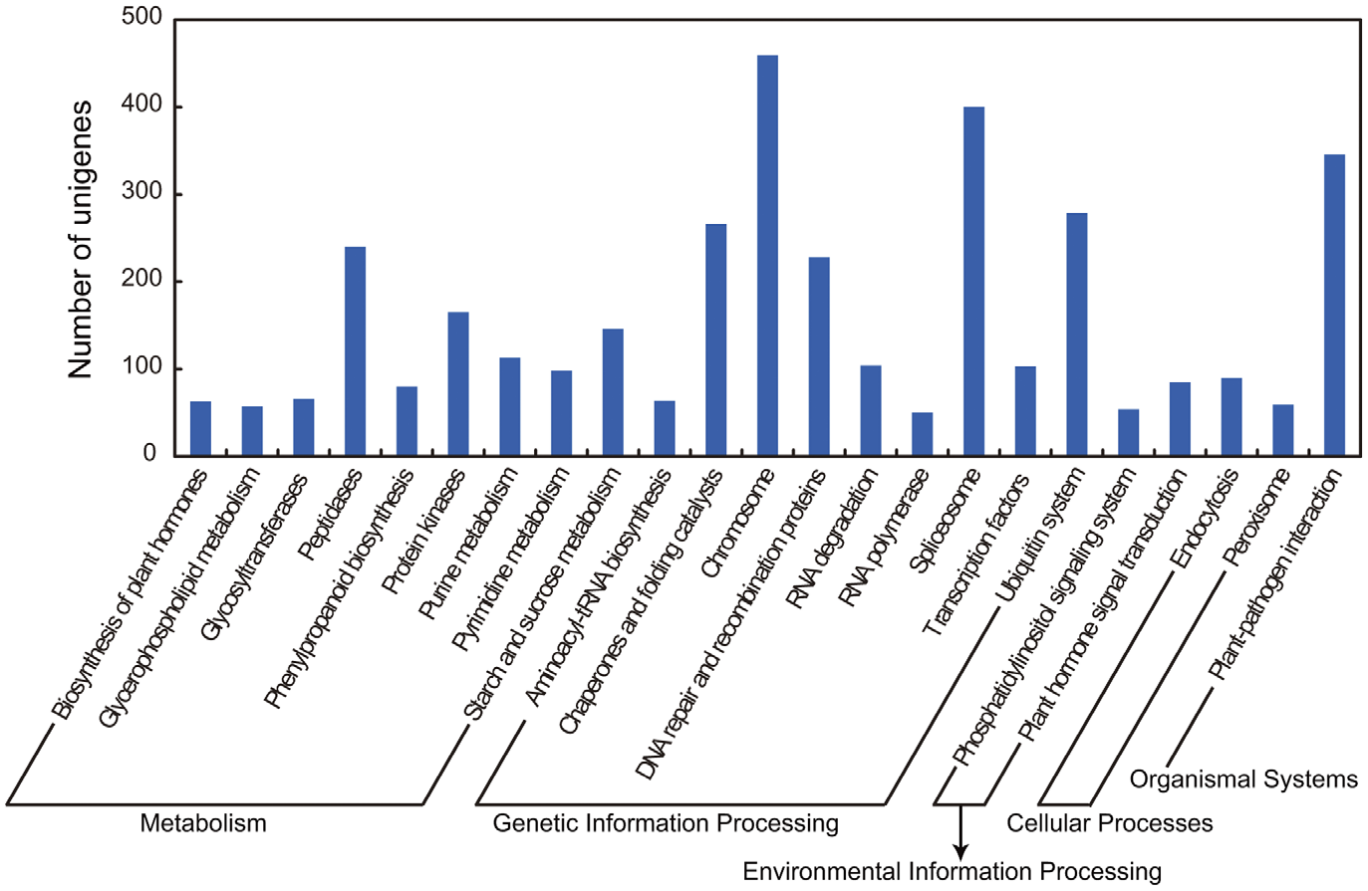
KEGG annotation of new transcripts. The new transcripts were distributed in 60 KEGG functional classes (Release 58.1, June 1, 2011), and only the classes with new transcripts number larger than 50 were shown in the figure.

We next compared our data with the existing unigene sequences of tobacco by BLAST analysis against the unigene sequences of tobacco from UniGene database of NCBI (http://www.ncbi.nlm.nih.gov/unigene). 61,508 of our unigenes had BLAST results, and the remaining 34,408 unigenes without BLAST results were considered to be new transcripts. BLAST analysis against the protein databases nr, Swiss-Prot, KEGG, COG and GO revealed that 16,052 of the new transcripts had BLAST results in those databases, and 5,076 transcripts had KEGG annotations. Among the new transcripts with KEGG annotations, 757 transcripts were unclassified and the remaining ones were distributed in 60 KEGG functional classes. The top three KEGG functional classes containing the largest numbers of new transcripts were chromosome, spliceosome and plant-pathogen interaction (Figure 2).

### Digital Gene Expression (DGE) Profiles

To investigate global gene expression changes after virus infection, individual DGE tag libraries were constructed from the 12 total RNA samples isolated from the infected and mock samples and sequenced by Illumina HiSeq 2000. Approximately five million raw tags were generated for each DGE library, and more than 90% of the raw tags in each library were clean tags. The statistics of DGE tags is shown in Table S1. To evaluate the normality of the dataset, the distribution of clean tag copy numbers was evaluated. In this analysis, the ‘total’ number of clean tags is the sum of all the clean tags and the number of ‘distinct’ clean tags is the number of different clean tags. In each library, more than 65% of the total clean tags were tags with copy numbers greater than 100, however these highly-expressed tags only represented 3.5–5.5% of the distinct clean tags in different samples. In contrast, 60% of the distinct clean tags had copy numbers between 2 and 5 but only represented around 5% of the total number of clean tags. These results reflect the principle that a small number of mRNAs are expressed at a very high abundance [20], while the majority are expressed at a very low level, and indicate that our DGE dataset was normally distributed.

**Figure 3:**
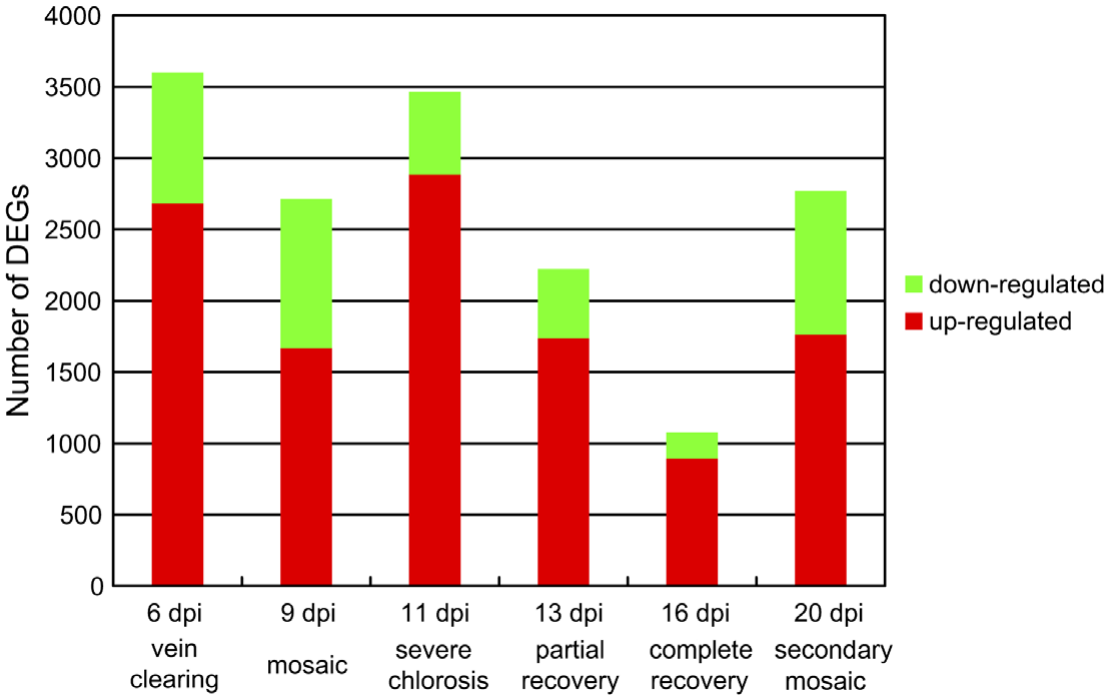
Differentially-expressed genes (DEGs) in different symptom stages.

We first compared pairs of DGE profiles of the infected and mock control samples collected at the same time to identify differentially-expressed genes (DEGs). DGE-tags were detected for 37,001 of the 56,768 unigenes that have the CATG sites required for library construction. We found that only 8,513 (23%) of the 37,001 unigenes were DEGs in at least one of the symptom stages, indicating that most of the 37,001 tag-mapped unigenes were expressed consistently during the course of disease development. Numbers of the identified DEGs differed in each stage of the infection (Figure 3). Approximately 3,500 host genes altered expression and most showed up-regulation at Stage 1 and Stage 3 when the infected leaves displayed vein clearing and severe chlorosis, respectively. By comparison, the lowest number of DEGs was detected in the “recovered” leaves at Stage 5, suggesting the least impact of virus infection on host gene expression in the leaves that appear essentially healthy. Top 20 genes that were up- or down-regulated and the KEGG pathways significantly enriched at different infection stages were listed in Table S2, S3, S4, S5, S6 and S7.

### Common Differentially Expressed Genes in Six Symptom Stages

Further analysis revealed that 150 unigenes showed differential expression at all of the six infection stages as compared to mock inoculation controls. Among these 150 common DEGs, 138 were up-regulated, and 10 were down-regulated in all of the six stages, which is similar to the pattern of total DEGs. We next analyzed the putative function of the common DEGs to determine if they encode conserved biological processes. According to the BLAST results, only 32 of these 150 unigenes had KEGG function annotations (Table S8). We found that 1 of the 32 annotated common DEGs related to genetic information processing (glutaredoxin) was down-regulated in all of the six stages. Among the 31 up-regulated common DEGs, 20, 6, 3 and 2 were putatively involved in metabolism, signaling, genetic information processing and in plant-pathogen interaction process of organismal systems, respectively (Table S8).

### Important KEGG Pathways Influenced by CMV Infection

The KEGG pathway analysis failed to identify any common KEGG pathway that was significantly enriched in all of the six infection stages. However, three pathways, namely ‘ribosome’ and ‘biosynthesis of secondary metabolites’ were significantly enriched in five of the infection stages with an exception only at complete recovery stage. The great majority of the significantly enriched KEGG pathways were related to metabolism and most of these metabolic pathways were enhanced by CMV infection, whereas pathways related to photosynthesis (‘photosynthesis’ and ‘carbon fixation in photosynthetic organisms’) and plant pigment metabolism (‘porphyrin and chlorophyll metabolism’, ‘carotenoid biosynthesis’ and ‘anthocyanin biosynthesis’) were inhibited at several symptom development stages. Of the remaining six pathways not correlated to metabolism, 4 pathways (‘ribosome’, ‘DNA replication’, ‘SNARE interactions in vesicular transport’ and ‘protein processing in endoplasmic reticulum’) relate to genetic information processing, 1 (‘Peroxisome’) to cellular processes, and 1 (‘Plant-pathogen interaction’) to organismal systems. The summary of important KEGG pathways influenced by M-CMV infection was shown in Table 3.

**Table 3:** Important KEGG pathways influenced by M-CMV infection.

Pathway	Enriched P-value*					
	6 dpi	9 dpi	11 dpi	13 dpi	16 dpi	20 dpi
Ribosome	9.28E-04	2.35E-04	7.12E-03	1.55E-02		1.12E-02
Biosynthesis of secondary metabolites	2.79E-03	1.70E-02	2.06E-05	2.35E-04		9.22E-05
Photosynthesis	1.32E-03	5.86E-03				
Carbon fixation in photosynthetic organisms	4.42E-02	1.43E-02				1.98E-03
Porphyrin and chlorophyll metabolism	1.74E-03		4.44E-02			1.25E-02
Carotenoid biosynthesis	7.47E-03				5.85E-03	
Anthocyanin biosynthesis	2.86E-02					2.71E-03
Plant-pathogen interaction				4.29E-03	2.42E-06	

* Only the P-values for pathways considered as significantly enriched (P-value<0.05) were shown in the table.

### DEGs and Pathways Related to the Initial Pathogenesis Process

**Figure 4:**
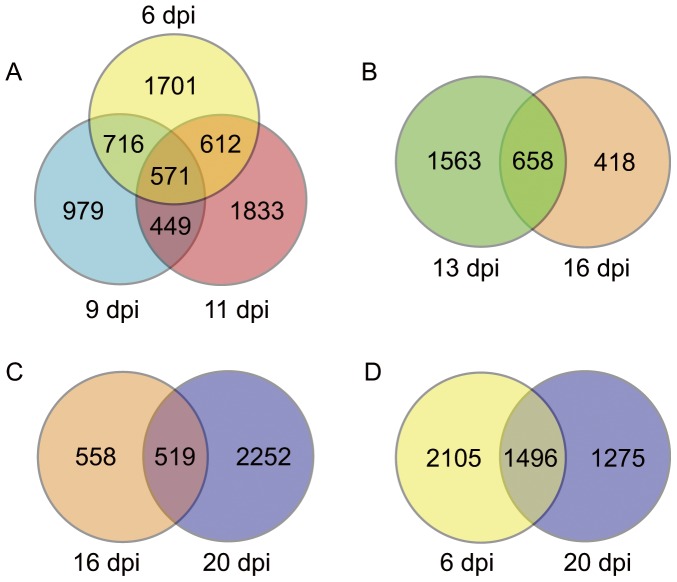
Relationship of DEGs in different symptom stages. A, relationship between DEGs at 6, 9 and 11 dpi; B, relationship between DEGs at 13 and 16 dpi; C, relationship between DEGs at 16 and 20 dpi; D, relationship between DEGs at 6 and 20 dpi.

The first pathogenesis process includes induction of vein clearing and development of mosaic and severe chlorosis symptoms between 6 and 11 dpi. We identified 571 common DEGs for these 3 infection stages, which included 430 and 80 that were up- and down-regulated, respectively (Figure 4A). As shown in Table S9, 115 common DEGs had KEGG annotation, and included 64, 19, 13 and 7 with KEGG annotation in metabolism, genetic information processing, signaling and plant-pathogen interaction process of organismal systems, respectively. Most of the annotated DEGs were up-regulated during the first pathogenesis and the down-regulated DEGs were related to energy metabolism (Sulfotransferase, V-type H+-transporting atpase subunit I, photosystem I subunit VIII and phosphoribulokinase), secondary metabolism (xanthoxin dehydrogenase, UDP-glucosyl transferase, casbene synthase, uroporphyrinogen decarboxylase, protochlorophyllide reductase, glutamine amidotransferase and anthocyanin 5-O-glucosyltransferase).

Based on the KEGG pathway analysis, the biological processes influenced most significantly during the the pathogenesis process were metabolism and genetic information processing. Energy metabolism (including the ‘sulfur metabolism’, ‘photosynthesis’ and ‘carbon fixation in photosynthetic organisms’ pathways) and pigment metabolism (including ‘porphyrin and chlorophyll metabolism’, ‘carotenoid biosynthesis’ and ‘anthocyanin biosynthesis’) were suppressed by virus infection. Several up-regulated pathways were related to the metabolism of terpenoids and polyketides (e.g. ‘zeatin biosynthesis’ and ‘diterpenoid biosynthesis’), which might regulate plant defense [34]. In line with results from previous studies [7], [11], we found that carbohydrate metabolism, amino acid metabolism, lipid metabolism and the biosynthesis of other secondary metabolites were also affected by M-CMV infection.

### DEGs and Pathways Related to the Recovery Process

There were 658 DEGs common for the partial recovery and complete recovery stages of the infection (Figure 4B), 574 of which were up-regulated in both stages. As shown in Table S10, 137 common DEGs had KEGG annotation, and included 65, 26, 20 and 15 DEGs related to metabolism, genetic information processing, signaling and plant-pathogen interaction process of organismal systems, respectively. Besides DEGs involved in plant-pathogen interactions, many DEGs (several chitinase, several protein kinases, several EREBP-like factors and several ubiquitin-related enzymes) have been previously implicated in the R-gene mediated plant defense process [35]–[38].

According to KEGG pathway analysis, there were 4 common KEGG pathways significantly enriched in both partial recovery and complete recovery stages. Three of them, namely ‘Monoterpenoid biosynthesis’, ‘Flavonoid biosynthesis’, ‘Cysteine and methionine metabolism’, were related to metabolism with the remaining one involved in ‘plant-pathogen interaction’ pathway. Notably, the ‘plant-pathogen interaction’ pathway was significantly enriched only in partial recovery and complete recovery stages, indicating genes of this pathway may contribute to symptom recovery.

### DEGs and Pathways Related to the Secondary Pathogenesis Process

To reveal the molecular processes of the secondary pathogenesis process, we compared the DEGs identified at 20 dpi with those detected at 16 dpi and 6 dpi, respectively (Figure 4C and 4D). There were 519 common DEGs for 20 dpi and 16 dpi, 433 of which were up-regulated in both stages. As shown in Table S11, 102 common DEGs had KEGG annotation, and included 52, 23, 14 and 7 related to metabolism, genetic information processing, signaling and plant-pathogen interaction process of organismal systems, respectively. The number of common DEGs identified at 20 dpi and 6 dpi was 1,496, which was the largest between any two stages. 1033 of common DEGs were up-regulated in both stages. As shown in Table S12, 279 common DEGs had KEGG annotation, and included 153, 66, 24 and 12 DEGs related to metabolism, genetic information processing, signaling and plant-pathogen interaction process of organismal systems, respectively.

During the secondary pathogenesis process (after 18 dpi), pathways that were significantly enriched were related to metabolic and genetic information processing. Several pathways were the same as those of vein clearing stage, while other pathways were the same as those of complete recovery stage, indicating that the secondary pathogenesis process is a complex process involving several molecular processes similar to both the initial pathogenic process immediately after virus-infection and the recovery process.

### Comparison of DGE Tag Data with qRT-PCR Expression Patterns

**Figure 5:**
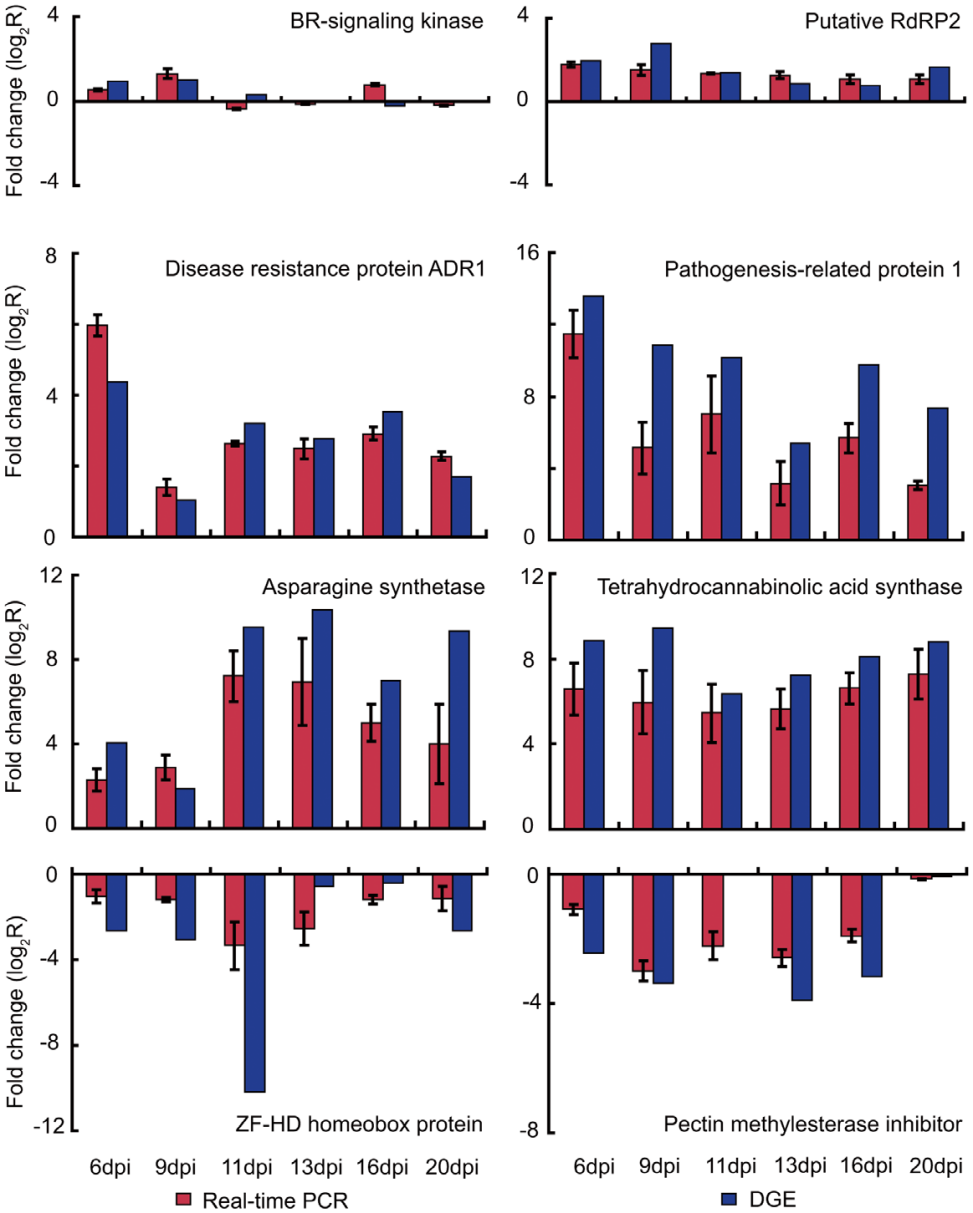
Quantitative RT-PCR (qRT-PCR) validation of the relative expression levels of transcripts selected from the DGE analysis. Expression profiles of selected genes as determined by qRT-PCR (Red) and DGE (Blue). The signal intensity of each transcript was normalized using EF1α. The y-axis shows the normalized expression level of the transcript. The x-axis indicates the number of days post infection (dpi). Error bars represent the standard deviations of qRT-PCR signals (n = 3).

In order to validate our DGE data, eight unigenes with annotations were selected for qRT-PCR analysis (Figure 5). The results showed that the qRT-PCR data of these genes were consistent with the DGE results. For example, both qRT-PCR and DGE analyses showed that genes encoding pathogenesis-related protein 1 (PR1), disease resistance protein ADR1, asparagine synthetase and tetrahydrocannabinolic acid synthase were significantly more highly expressed in M-CMV-infected tobacco leaves compared to uninfected leaves. Likewise, suppression of genes encoding the ZF-HD homeobox protein and pectin methylesterase inhibitor protein by M-CMV infection revealed by DGE analysis was verified by qRT-PCR analysis. We further analyzed expression of two genes encoding BR-signaling kinase and putative RNA-dependent RNA polymerase 2 (RdRP2) by qRT-PCR. These two genes were among those neither significantly up-regulated nor significantly down-regulated during M-CMV infection in the DGE dataset, which were also in agreement with our qRT-PCR expression patterns (Figure 5).

## Discussion

CMV has a world-wide distribution and is one of the most important plant pathogens [31], [32]. A previous study examined the resistance (R) gene-mediated resistance response of *A. thaliana* CMV by whole genome microarrays [5]. In this study, we used the next-generation sequencing approaches to investigate the gene expression changes associated with the characteristic disease development process induced in tobacco plants by M-CMV. RNA-Seq analysis identified a total of 95,916 unigenes in Xanthi tobacco, 34,408 of which were new transcripts compared to existing unigenes of tobacco (http://www.ncbi.nlm.nih.gov/unigene). KEGG pathway analysis showed that 20,978 of the unigenes could be grouped into 125 known pathways. The ‘metabolic pathways’ pathway contained the largest number of genes (4,965 unigenes), while the second largest pathway ‘plant-pathogen interaction’ included 1,633 annotated unigenes.

On the foundation of the unigene reference dataset, our DGE analysis further provided insights on the molecular mechanism for systemic symptom development of tobacco plants after CMV infection. Analysis on differentially expressed genes (DEGs) and significantly enriched KEGG pathways in different symptom stages indicated that several biological processes are influenced by virus infection. The most affected process is the metabolism process, as most of the annotated DEGs and enriched KEGG pathways are correlated to metabolism. The other biological processes significantly affected by CMV infection during the symptom development include photosynthesis, pigment metabolism and plant-pathogen interaction.

In previous studies, it was reported that genes related to photosynthesis and pigment metabolism were suppressed by virus infection [15]. In our study, we observed that many genes in pathways related to photosynthesis and pigment metabolism (‘photosynthesis’, ‘carbon fixation in photosynthetic organisms’, ‘porphyrin and chlorophyll metabolism’, ‘carotenoid biosynthesis’ and ‘anthocyanin biosynthesis’) were down-regulated during the initial pathogenesis process (from vein clearing stage to mosaic stage) and secondary pathogenesis process (secondary mosaic stage). We further showed that most of these pathways were neither significantly elevated nor suppressed during the recovery process. Our results suggest that these pathways, especially those related to pigment metabolism, may be directly responsible for the disease development. In addition, viral concentration analysis indicated that M-CMV concentration may also be correlated to symptom development of M-CMV-infected tobacco leaves.

Many genes in the ‘plant-pathogen interaction’ pathway were up-regulated during the symptom development process. These included genes involved in defense-related gene induction and innate immunity such as activation of genes coding for WRKY transcription factor and pathogenesis-related protein 1 reported previously [5], [7], [9]. Our results further revealed that several genes related to innate immunity, e.g., RIN4, ADR1 and RPM1 [37]–[39], were also induced during systemic symptom development. Interestingly, genes related to innate immunity such as RPS2, PBS1, RPS5, RIN4 and RPM1 [37], [38], were up-regulated from the severe chlorosis stage to the complete recovery stage, indicating that the innate immunity process was significantly enhanced during the transient recovery process.

Plant recovery is a phenomenon that following a symptomatic infection, newly emerged leaves and tissues are symptom-free and resistant to secondary infection by the same or a related virus. Plant recovery has been reported to be associated with the induction of antiviral RNA silencing and is considered as a possible consequence of RNA-based antiviral immunity [38], [40]–[42]. In this study, tobacco plants also recover from M-CMV infection and produce symptom-free leaves, which however later become symptomatic. Thus, the transient recovery process induced by M-CMV is quite different from the complete recovery observed in previous studies. Perhaps as a consequence, gene expression analysis revealed that the plant innate immunity process but not RNA-silencing process is activated during the transient recovery process. This suggests that the plant innate immunity process may play a key role in the transient recovery process. In addition, metabolic processes may also be important since pathways related to amino acid metabolism and secondary metabolism were elevated during the transient recovery process.

In summary, using mRNA sequencing and analysis of differential expression during six disease induction stages, we have provided a genome-wide transcript profile for systemic symptom development in tobacco plants infected with M-CMV. Although the molecular functions of some genes and their associated pathways remain largely unknown, this study provides insights into the molecular mechanisms of the tobacco symptom development process (especially the transient recovery and secondary pathogenesis processes) after M-CMV infection, and will facilitate further investigations of the detailed mechanisms of plant responses to viral infection.

## Materials and Methods

### Plant Growth and Virus Inoculation

Tobacco plants (*Nicotiana tabacum* cv. Xanthi nc) were grown in a greenhouse on a cycle of 16 h light at 30°C and 8 h dark at 25°C. Approximately 0.5 g systemically infected leaf tissue from M-CMV infected plants was harvested and immediately homogenized with kieselguhr in 1 ml water to prepare the virus inoculum. The viral inoculum was rub-inoculated onto the top two leaves of tobacco plants having four fully expanded leaves (Figure S5). The mock inoculum was prepared from leaves of healthy plants and applied in the same way as viral inoculum. 180 and 120 tobacco plants were virus-inoculated and mock-inoculated, repectively.

### RNA Extraction

Systemically infected leaves and leaves of mock-inoculated plants were harvested at 6, 9, 11, 13, 16 and 20 dpi as shown in Figure S5. In order to eliminate the variation between individual plants, five leaves from five different plants were mixed to prepare every RNA sample. The leaves were sampled from different plant individuals for each time point. The collected leaf tissues were frozen in liquid nitrogen and stored at −80°C. Total RNA was extracted from leaf tissue using TRIzol® Reagent (Invitrogen) following the manufacturer’s instructions and then treated with DNase I (Invitrogen). RNA concentration and integrity were analyzed on an Agilent 2100 Bioanalyzer (Agilent Technologies).

### Western Blot Analysis

Total proteins were extracted from tobacco leaves according to Mossop et al [43]. The protein concentrations were determined according to Bradford method [44]. The extracted total proteins were separated by tricine SDS polyacrylamide gel electrophoresis (Tricine-SDS-PAGE) [45], and the separated proteins were transferred onto a polyvinylidene difluoride (PVDF) membrane. The membrane was blocked overnight in 5% dry milk in TBS (20 mM Tris, PH 7.5; 150 mM NaCl) at 4°C. The membrane was incubated with primary antibodies (monoclonal antibody against the CMV CP) at room temperature for 2 h, followed by incubation with horseradish peroxidase-conjugated secondary antibody (Sigma) at room temperature for 1.5 h. ECL Plus Western blotting (GE Healthcare) detection were performed according to manufacture’s instruction.

### Library Construction, Sequencing and Data Analysis for RNA-Seq

Equal quantities of total RNA from six infected samples and six mock-inoculated samples were mixed to prepare the pooled RNA sample for RNA-Seq. The library for sequencing was prepared using the Illumina mRNA-Seq 8-Sample Prep Kit and the Illumina Sequencing Chip (flowcell) following the manufacturer’s instructions. Poly(A)-containing mRNA was isolated using magnetic beads with oligo(dT) and fragmented into short pieces. These short fragments were used as templates to synthesize first-strand cDNA using reverse transcriptase and random hexamer-primers. The second-strand cDNA was then synthesized using DNA polymerase I, dNTPs and RNase H. After purification and end repair, the cDNA fragments were ligated to sequencing adapters. Then fragments of a suitable size were purified and amplified by PCR to obtain the final library. The library was sequenced using an Illumina HiSeq™ 2000 and the raw reads generated by Solexa/Illumina sequencing were submitted to GEO database, Accession No. GSM746931.

Clean reads were obtained after removing reads which contained adaptor sequences, reads in which more than 10% of the bases were unknown, and reads in which more than half of the quality values of the bases were less than 5. Unigenes were obtained after transcriptome *de novo* assembly using SOAPdenovo [46]. Unigenes were analyzed by searching the protein databases nr, Swiss-Prot, KEGG and COG using BLASTX (E-value <0.00001). If results obtained using different databases conflicted with each other, the sequence direction of unigenes was decided based on a priority order of nr, Swiss-Prot, KEGG and COG. Information obtained from BLAST was used to extract CDS from unigene sequences and translate them into peptide sequences. Unigenes with no hits in BLAST were analysed with ESTScan [47] to predict their coding regions and decide their sequence direction. unigenes with nr annotation were further analyzed with Blast2go [48] to obtain GO annotations, and were then further classified according to GO functions using WEGO [49].

### Library Construction, Sequencing and Data Analysis for Digital Gene Expression (DGE)

Twelve individual tag libraries of samples (six infected samples and six mock-inoculated samples) were constructed. Sequence tags were prepared using an Illumina Gene Expression Sample Prep Kit and the Illumina Sequencing Chip (flowcell) following the manufacturer’s instructions. Magnetic beads with oligo(dTs) were used to purify poly(A)-containing mRNA and oligo-dT primers were used to sythesize double-stranded cDNA. The bead-bound cDNAs were then digested by endonuclease *Nla*III, which recognizes and cuts CATG sites, to generate 5' sticky ends. The cut-off fragments were washed away and Illumina adaptor 1 was ligated to the bead-bound fragments via the 5' sticky ends. The adaptor 1-ligated bead-bound fragments were then digested by *Mme*I, which cuts 17 bp downstream from the CATG site. The tags with adaptor 1 were purified and ligated to Illumina adaptor 2 via the 3' end of the tag. After 15 cycles of linear PCR amplification, 95 bp fragments were purified by 6% TBE polyacrylamide gel electrophoresis to obtain the final tag libraries. Sequencing was performed using an Illumina HiSeq™ 2000. Millions of raw reads with a sequencing length of 35 bp were generated. The raw tag data were deposited in GEO (NCBI), Accession Nos. GSM746919, GSM746920, GSM746921, GSM746922, GSM746923, GSM746924, GSM746925, GSM746926, GSM746927, GSM746928, GSM746929, GSM746930.

**Table 4:** Primer sets and PCR efficiency of unigenes selected in qRT-PCR.

Unigene ID	Gene name	Gene primers for qRT-PCR	PCR efficiency
17917	BR-signaling kinase	S*: AAGGTGATGTAGTGTTTTTGGGGT	1.93
		A**: CATCGCAAACTAAACTACTGAGGG	
21633	Putative RdRP2	S*: CAGCGGGACAACAGGAGGTATTTT	1.92
		A**: AAGTAACATGATACCACGCCGATG	
95370	ADR1	S*: GGACTGGTTCAGAATGGATTGCCC	2.00
		A**: AGTCGGACAGGTGAGTGACGGATA	
44065	PR1	S*: TCTCAACAAGACTATTTGGATGCC	1.97
		A**: GCATAGGCTGCTACCTGGTCGTCC	
18588	Asparagine synthetase	S*: GAGCGAGTGTGGCGTGTAGC	1.99
		A**: CCCATTAGCCATAGCAGGTTCA	
4211	Tetrahydrocannabinolic acid synthase	S*: TAGCACAGTGGAATGAAGAGGACG	1.94
		A**: CAGCATCAACAGAGGAAGAAGGAC	
11467	ZF-HD homeobox protein	S*: AGATGCTCTAAAATGTGCTGCTTG	1.96
		A**: TCTCCTTTTGGTCTTGTGTGAACT	
95361	Pectin methylesterase inhibitor protein	S*: GGCTCGTGCTGCTTTATCAGTTAG	1.98
		A**: GCAGTCCTTTACGGCTTGTTTTTC	
	EF1α	S*: TCGCCTTGTGGAAGTTTGAGAC	1.99
		A**: CACCAACAGCAACAGTTTGACG	

* S, sense primer;

** A, antisense primer.

The adaptors, empty tags (no tag sequence between the adaptors), low quality tags (tags containing one or more unknown nucleotides “N”) and tags with a copy number of 1 were removed from the raw data to obtain 21 bp clean tags. All clean tags were mapped to the transcriptome reference database generated by RNA-Seq. The number of unambiguous tags corresponding to each gene was calculated and normalized to the TPM (number of transcripts per million clean tags) to analyze the expression of different genes. To identify genes expressed differentially between the virus-infected samples and mock-inoculated samples, an algorithm was developed based on the method described by Audic and Claverie [50]. FDR (false discovery rate) was utilized to determine the threshold of P-values. "FDR ≤0.001 and the absolute value of the log2Ratio ≥1" were selected as the threshold for judging the diffrentially-expressed genes (DEGs).

In KEGG pathway analysis, the formula for calculating enriched P-values was:
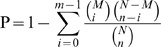



Here N is the total number of genes that had KEGG annotations, n is the number of differentially expressed genes in N, M is the total number of genes annotated to specific pathways, and m is number of differentially expressed genes in M. A P-value of 0.05 was selected as the threshold for deciding whether a gene set was significantly enriched.

### Quantitative Real-time PCR (qRT-PCR) Validation

In order to validate the DGE results, qRT-PCR analysis was performed using 3 RNA samples; the RNA sample used in the DGE experiment and two other replicates from different plant samples. Total RNA samples were extracted as described above, and 5 µg of each RNA sample was digested with 10 U of RNase-free DNase I (Invitrogen) for 30 min at 25°C. Reverse transcription was performed in a 20 µl reaction system using the Superscript III first-strand synthesis system (Invitrogen) according to the manufacturer’s protocol. The unigenes in qRT-PCR validation were selected randomly and the results of eight unigenes with annotations were shown. The primer sets were designed using Primer Premier 5.0 software (PREMIER Biosoft International) within the conserved region of nucleotide sequences aligned with numerous homologous sequences of plant found on NCBI (Table 4), and the parallel reactions using EF1α primers [51] were performed to normalize the amount of template cDNA added to each reaction. qRT-PCR was performed using a DyNAmo™ SYBR® Green qPCR kit (New England Biolab) according to the manufacturer’s instructions. qRT-PCR cycles were carried out on a Rotor-Gene RG3000A detection system (Corbett Research) as follows: 10 min at 95°C for denaturation, followed by 42 cycles of 10 s at 94°C for denaturation, 20 s at 58°C for annealing and 30 s at 72°C for extension. Fluorescence data was collected at 78°C. After a final extension at 72°C for 5 min, a melting curve was performed from 70 to 95°C (held for 1 s per 0.1°C increase) to examine the specificity of the amplified product. All qRT-PCR experiments were performed in triplicate using the independent samples mentioned above. Expression quantification and data analysis were performed in accordance with Bustin et al [52].

## Supporting Information

Figure S1 **Viral concentrations in leaves of different symptom stages.** Viral concentrations were estimated by western blot using actin as internal control. The control sample was prepared from healthy leaves.
(TIF)

Figure S2 **Unigene size distribution.**
(TIF)

Figure S3 **Unigene COG annotations.** Unigenes aligned to the COG database were grouped into 24 functional classes.
(TIF)

Figure S4 **Unigene GO annotations.** Unigenes with GO annotations (Release in June, 2011) were classified into three major functional categories: biological process, cellular components, and molecular functions.
(TIF)

Figure S5 **Schematic representation of virus inoculation and leaf collection.** Leaf 3 and Leaf 4 were leaves which viral inoculum was inoculated on. Leaf 5 with vein clearing symptom was collected at 6 dpi, and Leaf 5 with mosaic symptom was harvested at 9 dpi. Leaf 6 with severe chlorosis symptom was collected at 11 dpi, and Leaf 6 with partial recovery symptom was collected at 13 dpi. Leaf 7 with complete recovery symptom was collected at 16 dpi. Leaf 8 with secondary mosaic symptom was collected at 20 dpi.
(TIF)

Table S1 **Tag analysis statistics.**
(XLS)

Table S2 **Top 20 up-regulated and down-regulated genes and significantly enriched KEGG pathways at 6 dpi.**
(XLS)

Table S3 **Top 20 up-regulated and down-regulated genes and significantly enriched KEGG pathways at 9 dpi.**
(XLS)

Table S4 **Top 20 up-regulated and down-regulated genes and significantly enriched KEGG pathways at 11 dpi.**
(XLS)

Table S5 **Top 20 up-regulated and down-regulated genes and significantly enriched KEGG pathways at 13 dpi.**
(XLS)

Table S6 **Top 20 up-regulated and down-regulated genes and significantly enriched KEGG pathways at 16 dpi.**
(XLS)

Table S7 **Top 20 up-regulated and down-regulated genes and significantly enriched KEGG pathways at 20 dpi.**
(XLS)

Table S8 **KEGG-annotated common DEGs in six symptom stages.**
(DOC)

Table S9 **KEGG-annotated common DEGs at 6 dpi, 9 dpi and 11dpi.**
(DOC)

Table S10 **KEGG-annotated common DEGs at 13 dpi and 16 dpi.**
(DOC)

Table S11 **KEGG-annotated common DEGs at 20 dpi and 16 dpi.**
(DOC)

Table S12 **KEGG-annotated common DEGs at 20 dpi and 6 dpi.**
(DOC)
