# Supplementary figures and images for: Transcriptome Analysis of Nicotiana tabacum Infected by Cucumber mosaic virus during Systemic Symptom Development
Source: PLoS One. 2012 Aug 28;7(8):e43447. doi: 10.1371/journal.pone.0043447 (PMC3429483; doi:10.1371/journal.pone.0043447)

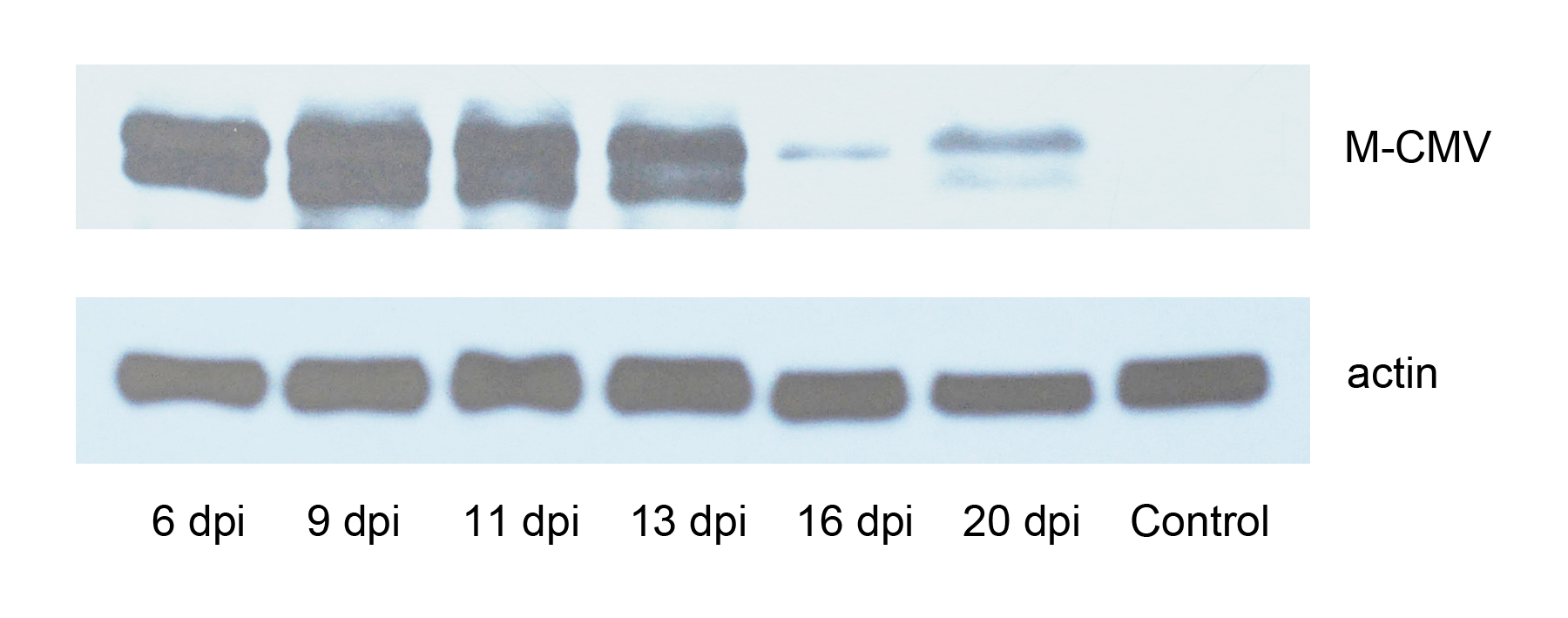

Supplement: Figure S1 — Viral concentrations in leaves of different symptom stages. Viral concentrations were estimated by western blot using actin as internal control. The control sample was prepared from healthy leaves. (TIF) [file pone.0043447.s001.tif]

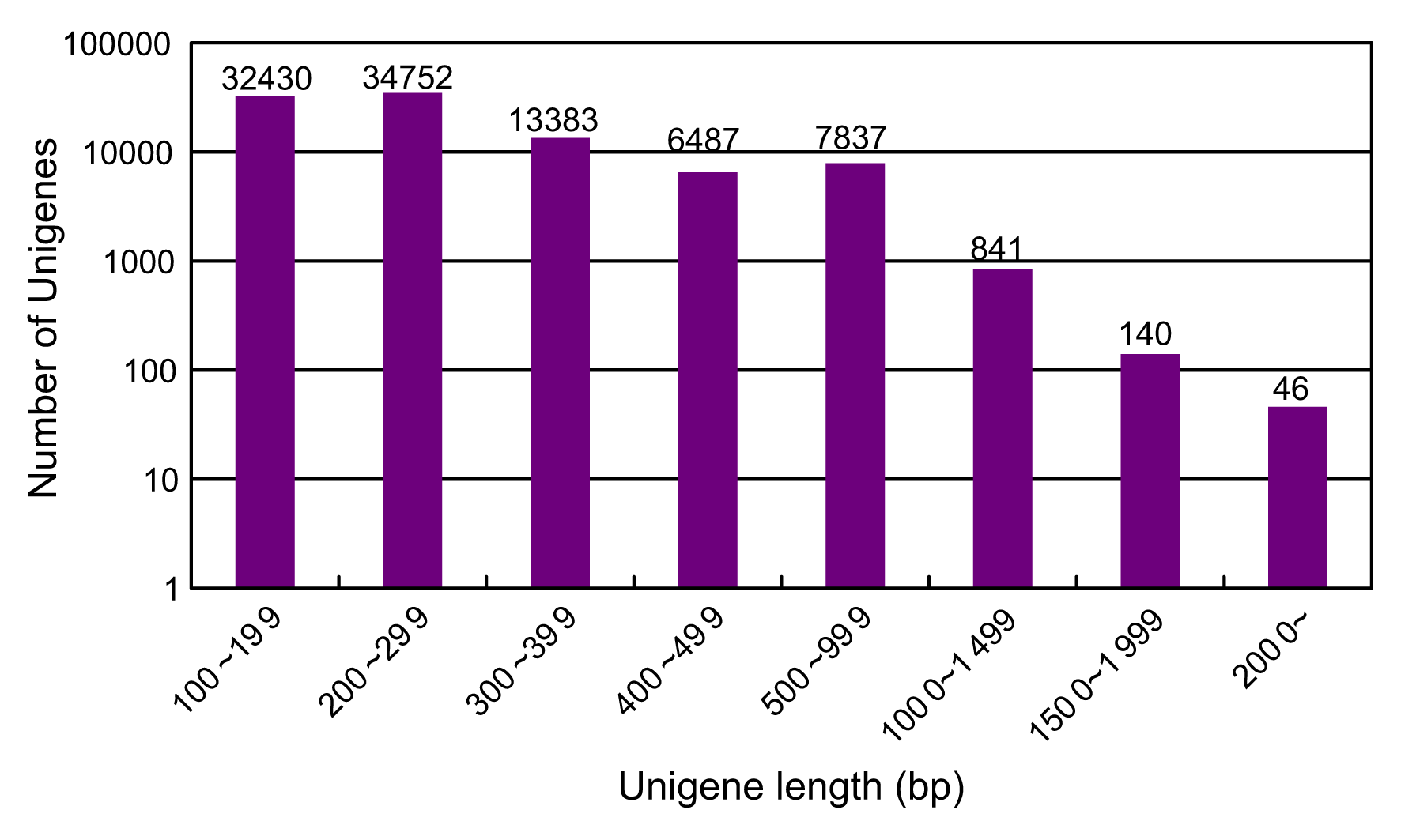

Supplement: Figure S2 — Unigene size distribution. (TIF) [file pone.0043447.s002.tif]

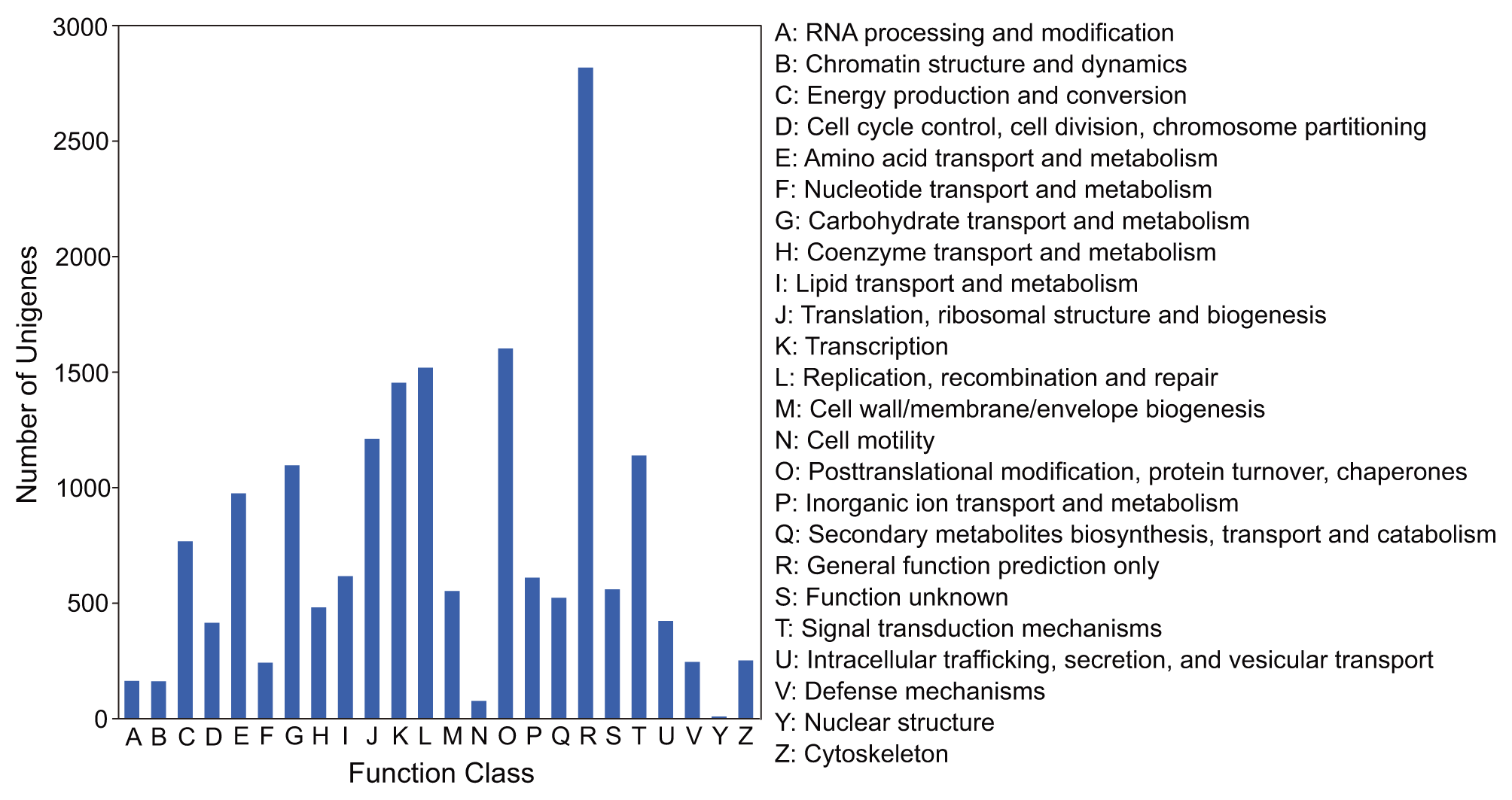

Supplement: Figure S3 — Unigene COG annotations. Unigenes aligned to the COG database were grouped into 24 functional classes. (TIF) [file pone.0043447.s003.tif]

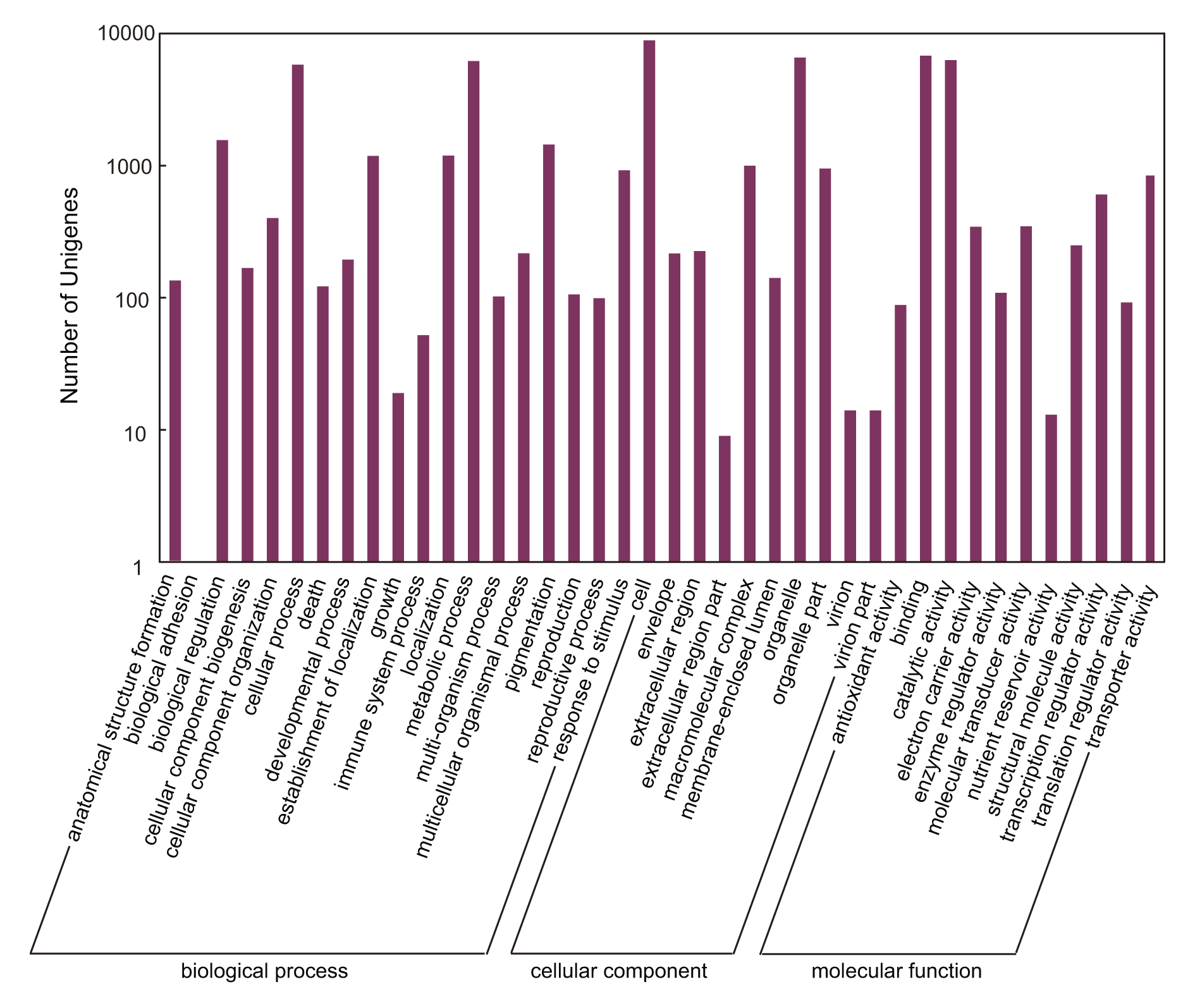

Supplement: Figure S4 — Unigene GO annotations. Unigenes with GO annotations (Release in June, 2011) were classified into three major functional categories: biological process, cellular components, and molecular functions. (TIF) [file pone.0043447.s004.tif]

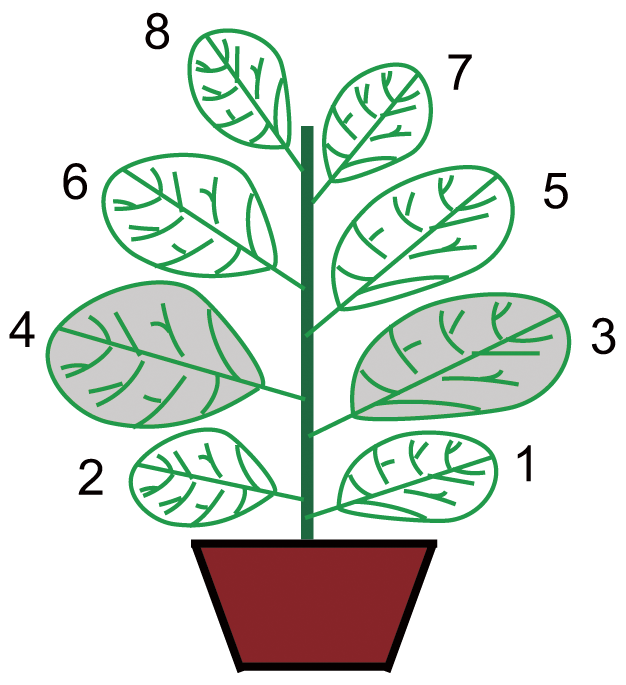

Supplement: Figure S5 — Schematic representation of virus inoculation and leaf collection. Leaf 3 and Leaf 4 were leaves which viral inoculum was inoculated on. Leaf 5 with vein clearing symptom was collected at 6 dpi, and Leaf 5 with mosaic symptom was harvested at 9 dpi. Leaf 6 with severe chlorosis symptom was collected at 11 dpi, and Leaf 6 with partial recovery symptom was collected at 13 dpi. Leaf 7 with complete recovery symptom was collected at 16 dpi. Leaf 8 with secondary mosaic symptom was collected at 20 dpi. (TIF) [file pone.0043447.s005.tif]
