# Supplementary material for: Transcriptome Analysis of Nicotiana tabacum Infected by Cucumber mosaic virus during Systemic Symptom Development
Source: PLoS One. 2012 Aug 28;7(8):e43447. doi: 10.1371/journal.pone.0043447 (PMC3429483; doi:10.1371/journal.pone.0043447)
Supplement: Table S8 — KEGG-annotated common DEGs in six symptom stages. (DOC) [file pone.0043447.s013.doc]

Table S8. KEGG-annotated common DEGs in six symptom stages.

| Unigene ID | Fold change (log_2_R) | | | | | | Putative function description | KEGG function class |
| --- | --- | --- | --- | --- | --- | --- | --- | --- |
|  | 6dpi | 9dpi | 11dpi | 13dpi | 16dpi | 20dpi |  |  |
| Unigene70534 | 2.72 | 2.11 | 2.49 | 2.7 | 1.96 | 1.84 | RIN4, RPM1 interacting protein 4 | Organismal Systems; Environmental Adaptation |
| Unigene94280 | 4.46 | 1.80 | 2.67 | 2.36 | 3.86 | 2.15 | Serine/threonine-protein kinase PBS1 | Organismal Systems; Environmental Adaptation |
| Unigene19450 | 2.54 | 1.50 | 1.81 | 1.53 | 1.9 | 1.89 | UDP-arabinose 4-epimerase | Metabolism; Carbohydrate Metabolism |
| Unigene48531 | 1.39 | 1.48 | 1.78 | 1.04 | 1.36 | 1.2 | Pyruvate kinase | Metabolism; Carbohydrate Metabolism |
| Unigene80198 | 3.48 | 2.51 | 2.46 | 2.11 | 3.51 | 1.93 | Glucan endo-1,3-beta-glucosidase | Metabolism; Carbohydrate Metabolism |
| Unigene41888 | 3.41 | 2.70 | 2.61 | 2.13 | 1.4 | 1.33 | Basic chitinase | Metabolism; Carbohydrate Metabolism |
| Unigene58623 | 1.51 | 2.23 | 2.53 | 3.45 | 1.87 | 1.33 | Endochitinase 3 | Metabolism; Carbohydrate Metabolism |
| Unigene63676 | 8.02 | 5.90 | 5.82 | 2.62 | 4.80 | 4.84 | Basic endochitinase | Metabolism; Carbohydrate Metabolism |
| Unigene84601 | 1.16 | 1.11 | 1.56 | 2.24 | 1.69 | 1.22 | Peroxisomal acyl-coenzyme A oxidase 1 | Metabolism; Lipid Metabolism Cellular Processes; Transport and Catabolism |
| Unigene94996 | 2.16 | 1.54 | 2.29 | 1.47 | 1.37 | 3.38 | Lipase-like protein | Metabolism; Lipid Metabolism |
| Unigene10873 | 2.81 | 3.76 | 3.40 | 4.70 | 2.09 | 1.69 | Saccharopine dehydrogenase | Metabolism; Amino Acid Metabolism |
| Unigene24728 | 2.19 | 1.48 | 3.96 | 3.39 | 2.94 | 2.11 | 1,2-dihydroxy-3-keto-5-methylthiopentene dioxygenase | Metabolism; Amino Acid Metabolism |
| Unigene85655 | 2.65 | 1.85 | 2.38 | 1.96 | 1.88 | 2.89 | Type 2 proly 4-hydroxylase | Metabolism; Amino Acid Metabolism |
| Unigene85968 | 1.66 | 1.62 | 3.64 | 3.96 | 3.7 | 3.70 | Tryptophan synthase beta chain 2 | Metabolism; Amino Acid Metabolism |
| Unigene85934 | 2.34 | 2.87 | 2.97 | 3.79 | 1.57 | 2.22 | Probable glutathione S-transferase | Metabolism; Metabolism of Other Amino Acid |
| Unigene92746 | 2.94 | 2.12 | 2.03 | 3.66 | 2.09 | 1.80 | Putative NADH dehydrogenase | Metabolism; Energy Metabolism |
| Unigene22886 | 3.17 | 1.13 | 1.75 | 1.88 | 2.82 | 3.95 | Cytochrome P450 CYP71D47v1 | Metabolism; Metabolism of Terpenoids and Polyketides Metabolism; Biosynthesis of Other Secondary Metabolites |
| Unigene83015 | 5.13 | 3.90 | 3.35 | 3.61 | 2.26 | 2.41 | Elicitor-inducible cytochrome P450 | Metabolism; Metabolism of Terpenoids and Polyketides Metabolism; Biosynthesis of Other Secondary Metabolites |
| Unigene64249 | 1.92 | 2.04 | 2.65 | 2.71 | 2.31 | 3.22 | 1-deoxy-D-xylulose-5-phosphate synthase | Metabolism; Metabolism of Terpenoids and Polyketides Metabolism; Biosynthesis of plant hormones |
| Unigene32838 | 3.95 | 2.71 | 1.99 | 2.70 | 1.48 | 2.35 | Cytochrome P450 CYP92A2v4 | Metabolism; Biosynthesis of Other Secondary Metabolites |
| Unigene5738 | 3.71 | 1.52 | 4.29 | 1.20 | 3.13 | 3.13 | Putative leucoanthocyanidin dioxygenase | Metabolism; Biosynthesis of Other Secondary Metabolites |
| Unigene94649 | 1.54 | 1.83 | 2.20 | 3.18 | 1.81 | 1.30 | Protein phosphatase 2C | Unclassified; Metabolism |
| Unigene19341 | 2.68 | 1.00 | 1.64 | 1.19 | 2.49 | 5.14 | Ethylene-responsive transcription factor | Genetic Information Processing; Transcription |
| Unigene23813 | 2.22 | 1.83 | 1.80 | 2.61 | 1.18 | 2.35 | Pre-mRNA-splicing factor | Genetic Information Processing; Transcription |
| Unigene25408 | 7.67 | 1.85 | 2.62 | 1.71 | 2.37 | 3.52 | Ubiquitin-protein ligase | Genetic Information Processing; Folding, Sorting and Degradation |
| Unigene17433 | -2.00 | -2.84 | -2.81 | -2.56 | -1.03 | -3.06 | Glutaredoxin | Genetic Information Processing; Folding, Sorting and Degradation |
| Unigene24337 | 1.73 | 1.79 | 2.53 | 1.53 | 2.83 | 2.67 | Aquaporin-like protein | Environmental Information Processing; Signaling Molecules and Interaction |
| Unigene94597 | 1.40 | 1.01 | 2.08 | 2.16 | 3.02 | 2.53 | Glutamate-gated kainate-type ion channel receptor subunit | Environmental Information Processing; Signaling Molecules and Interaction |
| Unigene16039 | 5.22 | 3.19 | 2.68 | 3.20 | 4.08 | 1.24 | Serine/threonine protein kinase family protein | Unclassified; Cellular Processes and Signaling |
| Unigene17628 | 3.21 | 2.43 | 2.34 | 2.68 | 2.24 | 2.86 | Putative serine/threonine-protein kinase-like protein | Unclassified; Cellular Processes and Signaling |
| Unigene25450 | 3.02 | 1.30 | 2.08 | 1.61 | 1.77 | 1.59 | S-locus-like receptor protein kinase | Unclassified; Cellular Processes and Signaling |
| Unigene25039 | 7.78 | 5.81 | 3.25 | 4.92 | 6.00 | 3.25 | Chloroplast nucleoid DNA binding protein | Unknown |
