# Supplementary material for: Transcriptome Analysis of Nicotiana tabacum Infected by Cucumber mosaic virus during Systemic Symptom Development
Source: PLoS One. 2012 Aug 28;7(8):e43447. doi: 10.1371/journal.pone.0043447 (PMC3429483; doi:10.1371/journal.pone.0043447)
Supplement: Table S9 — KEGG-annotated common DEGs at 6 dpi, 9 dpi and 11dpi. (DOC) [file pone.0043447.s014.doc]

Table S9. KEGG-annotated common DEGs at 6 dpi, 9 dpi and 11dpi.

| Gene | Fold change (log_2_R) | | | Putative function description | KEGG function class |
| --- | --- | --- | --- | --- | --- |
|  | 6 dpi | 9 dpi | 11 dpi |  |  |
| Unigene11449 | 1.28 | 1.62 | 1.06 | Beta-galactosidase | Metabolism; Carbohydrate Metabolism Metabolism; Glycan Biosynthesis and Metabolism |
| Unigene19450 | 2.54 | 1.5 | 1.81 | UDP-arabinose 4-epimerase | Metabolism; Carbohydrate Metabolism |
| Unigene2493 | 1.06 | 1.94 | 1.98 | Class IV chitinase | Metabolism; Carbohydrate Metabolism |
| Unigene3219 | 2.99 | 3.13 | 2.19 | Stachyose synthetase | Metabolism; Carbohydrate Metabolism |
| Unigene41888 | 3.41 | 2.7 | 2.61 | Basic chitinase | Metabolism; Carbohydrate Metabolism |
| Unigene48531 | 1.39 | 1.48 | 1.78 | Pyruvate kinase | Metabolism; Carbohydrate Metabolism |
| Unigene58623 | 1.51 | 2.23 | 2.53 | Endochitinase 3 | Metabolism; Carbohydrate Metabolism |
| Unigene63676 | 8.02 | 5.9 | 5.82 | Basic endochitinase | Metabolism; Carbohydrate Metabolism |
| Unigene64448 | 1.95 | 1.63 | 2.11 | Pectinesterase | Metabolism; Carbohydrate Metabolism |
| Unigene80198 | 3.48 | 2.51 | 2.46 | Glucan endo-1,3-beta-glucosidase | Metabolism; Carbohydrate Metabolism |
| Unigene95307 | 2.71 | 1.59 | 2.22 | Beta-fructofuranosidase | Metabolism; Carbohydrate Metabolism |
| Unigene24090 | -1.97 | -2.72 | -1.58 | Serine O-acetyltransferase | Metabolism; Amino Acid Metabolism Metabolism; Energy Metabolism |
| Unigene95235 | 2.73 | 1.34 | 1.59 | Urease | Metabolism; Amino Acid Metabolism Metabolism; Nucleotide Metabolism |
| Unigene61912 | -1.06 | -1.25 | -1.72 | Sulfotransferase | Metabolism; Amino Acid Metabolism Metabolism; Biosynthesis of Other Secondary Metabolites |
| Unigene10873 | 2.81 | 3.76 | 3.4 | Saccharopine dehydrogenase | Metabolism; Amino Acid Metabolism |
| Unigene18588 | 4.06 | 1.88 | 9.52 | Asparagine synthetase | Metabolism; Amino Acid Metabolism |
| Unigene24728 | 2.19 | 1.48 | 3.96 | 1,2-dihydroxy-3-keto-5-methylthiopentene dioxygenase | Metabolism; Amino Acid Metabolism |
| Unigene5488 | -1.94 | -2.37 | 1.51 | Phosphoserine phosphatase | Metabolism; Amino Acid Metabolism |
| Unigene70561 | 1.27 | 3.09 | 2.8 | Aspartate aminotransferase | Metabolism; Amino Acid Metabolism |
| Unigene85655 | 2.65 | 1.85 | 2.38 | Type 2 proly 4-hydroxylase | Metabolism; Amino Acid Metabolism |
| Unigene85968 | 1.66 | 1.62 | 3.64 | Tryptophan synthase beta chain 2 | Metabolism; Amino Acid Metabolism |
| Unigene24583 | 12.21 | 10.82 | 10.56 | Glutathione S-transferase | Metabolism; Metabolism of Other Amino Acids |
| Unigene4779 | 2.21 | 1.09 | 1.51 | Methionine S-methyltransferase | Metabolism; Metabolism of Other Amino Acids |
| Unigene54832 | 2.88 | 2.19 | 1.59 | Glutathione S-transferase | Metabolism; Metabolism of Other Amino Acids |
| Unigene94115 | 3.94 | 3.24 | 3.72 | Glutathione S-transferase | Metabolism; Metabolism of Other Amino Acids |
| Unigene85934 | 2.34 | 2.87 | 2.97 | Probable glutathione S-transferase | Metabolism; Metabolism of Other Amino Acids |
| Unigene95363 | 4.46 | 1.57 | 4.37 | Cytochrome P450 CYP72a55v2 | Metabolism; Lipid Metabolism Metabolism; Xenobiotics Biodegradation and Metabolism |
| Unigene84601 | 1.16 | 1.11 | 1.56 | Peroxisomal acyl-coenzyme A oxidase 1 | Metabolism; Lipid Metabolism Cellular Processes; Transport and Catabolism |
| Unigene94996 | 2.16 | 1.54 | 2.29 | Lipase-like protein | Metabolism; Lipid Metabolism |
| Unigene23411 | 1.28 | 1.13 | 1.27 | CTP synthase | Metabolism; Nucleotide Metabolism |
| Unigene13692 | -1.94 | -1.89 | -2.07 | Anthocyanin 5-O-glucosyltransferase | Metabolism; Glycan Biosynthesis and Metabolism Metabolism; Biosynthesis of Other Secondary Metabolites |
| Unigene88786 | 1.91 | 1.51 | 3.01 | Lysosomal alpha-mannosidase | Metabolism; Glycan Biosynthesis and Metabolism |
| Unigene89930 | 1.21 | 2.1 | -1.24 | UDP-glucose:glycoprotein glucosyltransferase | Metabolism; Glycan Biosynthesis and Metabolism |
| Unigene93634 | 1.27 | 1.04 | 1.16 | Callose synthase | Metabolism; Glycan Biosynthesis and Metabolism |
| Unigene9438 | 1.09 | 1.03 | 1.05 | Callose synthase | Metabolism; Glycan Biosynthesis and Metabolism |
| Unigene88993 | -1.97 | -1.15 | 2.09 | V-type H+-transporting atpase subunit I | Metabolism; Energy Metabolism Cellular Processes; Transport and Catabolism |
| Unigene75459 | -3.95 | -1.66 | -2.39 | Photosystem I subunit VIII | Metabolism; Energy Metabolism |
| Unigene87733 | -2.16 | -1.9 | -1.44 | Phosphoribulokinase | Metabolism; Energy Metabolism |
| Unigene92746 | 2.94 | 2.12 | 2.03 | Putative NADH dehydrogenase | Metabolism; Energy Metabolism |
| Unigene64249 | 1.92 | 2.04 | 2.65 | 1-deoxy-D-xylulose-5 -phosphate synthase | Metabolism; Metabolism of Terpenoids and Polyketides Metabolism; Biosynthesis of plant hormones |
| Unigene22886 | 3.17 | 1.13 | 1.75 | Cytochrome P450 CYP71D47v1 | Metabolism; Metabolism of Terpenoids and Polyketides Metabolism; Biosynthesis of Other Secondary Metabolites |
| Unigene83015 | 5.13 | 3.9 | 8.26 | Elicitor-inducible cytochrome P450 | Metabolism; Metabolism of Terpenoids and Polyketides Metabolism; Biosynthesis of Other Secondary Metabolites |
| Unigene10169 | 3.21 | 1.28 | 2.98 | Cytochrome P450-dependent fatty acid hydroxylase | Metabolism; Metabolism of Terpenoids and Polyketides Metabolism; Biosynthesis of Other Secondary Metabolites |
| Unigene2842 | -1.62 | -1.55 | 1.25 | Cytochrome P450 | Metabolism; Metabolism of Terpenoids and Polyketides Metabolism; Biosynthesis of Other Secondary Metabolites |
| Unigene11396 | 2.48 | 1.54 | 1.94 | Xanthoxin dehydrogenase | Metabolism; Metabolism of Terpenoids and Polyketides |
| Unigene2829 | -3.65 | -2.48 | -1.91 | Xanthoxin dehydrogenase | Metabolism; Metabolism of Terpenoids and Polyketides |
| Unigene33294 | -2.63 | -2.7 | -1.13 | Xanthoxin dehydrogenase | Metabolism; Metabolism of Terpenoids and Polyketides |
| Unigene35240 | -2.41 | -2.89 | -3.89 | UDP-glucosyl transferase | Metabolism; Metabolism of Terpenoids and Polyketides |
| Unigene87689 | 9.88 | 9.44 | 10.78 | Casbene synthase | Metabolism; Metabolism of Terpenoids and Polyketides |
| Unigene32315 | -2.4 | -1.02 | -1.82 | Uroporphyrinogen decarboxylase | Metabolism; Metabolism of Cofactors and Vitamins |
| Unigene40160 | -2.76 | -2.04 | -4.5 | Protochlorophyllide reductase | Metabolism; Metabolism of Cofactors and Vitamins |
| Unigene45545 | -1.87 | -1.12 | -1.12 | Glutamine amidotransferase | Metabolism; Metabolism of Cofactors and Vitamins |
| Unigene16795 | -1.51 | -1.3 | -2.63 | 2-oxoglutarate-dependent dioxygenase | Metabolism; Biosynthesis of Other Secondary Metabolites |
| Unigene25148 | 10.5 | 2.6 | 9.78 | 2-oxoglutarate-dependent dioxygenase | Metabolism; Biosynthesis of Other Secondary Metabolites |
| Unigene32838 | 3.95 | 2.71 | 1.99 | Cytochrome P450 CYP92A2v4 | Metabolism; Biosynthesis of Other Secondary Metabolites |
| Unigene5738 | 3.71 | 1.52 | 4.29 | Putative leucoanthocyanidin dioxygenase | Metabolism; Biosynthesis of Other Secondary Metabolites |
| Unigene35083 | -3.05 | -2.48 | -1.19 | Phytepsin | Metabolism; Enzyme Families |
| Unigene53640 | -1.75 | -1.23 | -1.7 | Phytepsin | Metabolism; Enzyme Families |
| Unigene91699 | 1.08 | -1.12 | 2.25 | Aminoacylase | Metabolism; Enzyme Families |
| Unigene12720 | 13.61 | 10.72 | 8.6 | Cysteine proteinase, putative | Unclassified; Metabolism |
| Unigene14651 | -2.75 | -2.4 | 1.42 | Dual specificity protein phosphatase | Unclassified; Metabolism |
| Unigene3899 | 1.82 | -1.15 | 1.55 | Ca2+-transporting atpase | Unclassified; Metabolism |
| Unigene93852 | 10.21 | 9.65 | 3.52 | Cysteine proteinase, putative | Unclassified; Metabolism |
| Unigene94649 | 1.54 | 1.83 | 2.2 | Protein phosphatase 2C | Unclassified; Metabolism |
| Unigene86427 | 1.88 | 1.13 | 1.54 | Large subunit ribosomal protein l7e | Genetic Information Processing; Translation |
| Unigene86759 | -1.22 | -1.31 | -1.24 | Large subunit ribosomal protein L10 | Genetic Information Processing; Translation |
| Unigene88400 | -1.85 | -3.06 | 1.6 | Elongation factor EF-G | Genetic Information Processing; Translation |
| Unigene16158 | 8.88 | 4.07 | 8.74 | EREBP (ethylene-responsive element binding protein)-like factor | Genetic Information Processing; Transcription |
| Unigene18502 | 1.47 | 1.09 | 1.07 | U4/U6.U5 tri-snrnp component SNU23 | Genetic Information Processing; Transcription |
| Unigene19341 | 2.68 | 1 | 1.64 | Ethylene-responsive transcription factor | Genetic Information Processing; Transcription |
| Unigene23813 | 2.22 | 1.83 | 1.8 | Pre-mrna-splicing factor | Genetic Information Processing; Transcription |
| Unigene50147 | 2.94 | 1.33 | 3.47 | RAV-like factor | Genetic Information Processing; Transcription |
| Unigene86613 | -2.24 | -1.14 | 1.61 | EREBP(ethylene-responsive element binding protein)-like factor | Genetic Information Processing; Transcription |
| Unigene88793 | 3.12 | 2.68 | 1.57 | EREBP(ethylene-responsive element binding protein)-like factor | Genetic Information Processing; Transcription |
| Unigene94363 | 1.07 | 1.22 | 1.23 | Probable DNA repair protein | Genetic Information Processing; Replication and Repair |
| Unigene17433 | -2 | -2.84 | -2.81 | Glutaredoxin | Genetic Information Processing; Folding, Sorting and Degradation |
| Unigene25408 | 7.67 | 1.85 | 2.62 | Ubiquitin-protein ligase | Genetic Information Processing; Folding, Sorting and Degradation |
| Unigene62779 | 1.86 | 1.41 | 1.25 | Transitional endoplasmic reticulum atpase | Genetic Information Processing; Folding, Sorting and Degradation |
| Unigene76164 | 2.61 | 1.26 | 1.23 | SKP1-like protein 3 | Genetic Information Processing; Folding, Sorting and Degradation |
| Unigene81714 | 2.14 | 1.21 | 1.01 | Luminal-binding protein 2 | Genetic Information Processing; Folding, Sorting and Degradation |
| Unigene93027 | 1.42 | 1.26 | 1.72 | Hsp70-interacting protein | Genetic Information Processing; Folding, Sorting and Degradation |
| Unigene56426 | 3.99 | 1.57 | 3.03 | Mitochondrial chaperone BCS1 | Unclassified; Genetic Information Processing |
| Unigene88419 | 8.74 | 8.72 | 8.16 | Mitochondrial chaperone BCS1 | Unclassified; Genetic Information Processing |
| Unigene25242 | 1.69 | 2.08 | 2.41 | ABC transporter B | Environmental Information Processing; Membrane Transport |
| Unigene24337 | 1.73 | 1.79 | 2.53 | Aquaporin-like protein | Environmental Information Processing; Signaling Molecules and Interaction |
| Unigene86373 | -1.55 | -1.99 | 1.55 | Aquaporin SIP | Environmental Information Processing; Signaling Molecules and Interaction |
| Unigene94597 | 1.4 | 1.01 | 2.08 | Glutamate-gated kainate-type ion channel receptor subunit | Environmental Information Processing; Signaling Molecules and Interaction |
| Unigene1262 | 3.43 | 1.82 | 1.58 | Protein brassinosteroid insensitive 1 | Environmental Information Processing; Signal Transduction |
| Unigene87665 | 2.62 | 1.46 | 1.45 | Probable serine/threonine-protein kinase drka | Environmental Information Processing; Signal Transduction |
| Unigene88910 | 2.38 | -1.29 | 1.74 | Extracellular signal-regulated kinase 1/2 | Environmental Information Processing; Signal Transduction |
| Unigene16039 | 5.22 | 3.19 | 2.68 | Serine/threonine protein kinase family protein | Unclassified; Cellular Processes and Signaling |
| Unigene17628 | 3.21 | 2.43 | 2.34 | Putative serine/threonine-protein kinase-like protein | Unclassified; Cellular Processes and Signaling |
| Unigene23060 | 1.53 | 1.33 | 1.31 | Serine/threonine protein kinase family protein | Unclassified; Cellular Processes and Signaling |
| Unigene25450 | 3.02 | 1.3 | 2.08 | S-locus-like receptor protein kinase | Unclassified; Cellular Processes and Signaling |
| Unigene87467 | -2.02 | -2.65 | 1.51 | Cytochrome c-type biogenesis protein ccmh | Unclassified; Cellular Processes and Signaling |
| Unigene91711 | 4.35 | 2.99 | 8.74 | Avr9/Cf-9 induced kinase 1 | Unclassified; Cellular Processes and Signaling |
| Unigene24690 | 2.21 | 1.43 | 3.1 | Probable LRR receptor-like serine/threonine-protein kinase | Unclassified; Cellular Processes and Signaling |
| Unigene66869 | 1.53 | 1.64 | 2.3 | Actin | Cellular Processes; Cell Motility |
| Unigene1306 | 10.33 | 2.57 | 3.81 | Interleukin-1 receptor-associated kinase 4 | Cellular Processes; Cell Growth and Death |
| Unigene15522 | 2.7 | 1.56 | 1.78 | Interleukin-1 receptor-associated kinase 4 | Cellular Processes; Cell Growth and Death |
| Unigene83363 | 2.51 | 1.08 | 1.61 | Interleukin-1 receptor-associated kinase 4 | Cellular Processes; Cell Growth and Death |
| Unigene95065 | 3.87 | 1.66 | 3.16 | Interleukin-1 receptor-associated kinase 4 | Cellular Processes; Cell Growth and Death |
| Unigene95757 | 3.72 | 1.58 | 2.09 | Interleukin-1 receptor-associated kinase 4 | Cellular Processes; Cell Growth and Death |
| Unigene38638 | 2.72 | 1.33 | 2.68 | WRKY transcription factor 33 | Organismal Systems; Environmental Adaptation |
| Unigene39638 | 11.99 | 5.09 | 8.74 | WRKY transcription factor 33 | Organismal Systems; Environmental Adaptation |
| Unigene44065 | 13.58 | 10.86 | 10.18 | Pathogenesis-related protein 1 | Organismal Systems; Environmental Adaptation |
| Unigene63748 | 11.21 | 3.26 | 3.64 | WRKY transcription factor 25 | Organismal Systems; Environmental Adaptation |
| Unigene70534 | 2.72 | 2.11 | 2.49 | RIN4, RPM1 interacting protein 4 | Organismal Systems; Environmental Adaptation |
| Unigene840 | 2.38 | 1.54 | 1.49 | Cyclic nucleotide gated channel | Organismal Systems; Environmental Adaptation |
| Unigene94280 | 4.46 | 1.8 | 2.67 | Serine/threonine-protein kinase PBS1 | Organismal Systems; Environmental Adaptation |
| Unigene2442 | 1.18 | 1.39 | 1.55 | Hypothetical protein | Unknown |
| Unigene25039 | 12.69 | 10.72 | 8.16 | Chloroplast nucleoid DNA binding protein | Unknown |
| Unigene25206 | 3.68 | 2.7 | 3.57 | SNF4, homolog of yeast sucrose nonfermenting 4 | Unknown |
| Unigene86202 | -1.26 | -1.03 | -1.14 | Hypothetical protein | Unknown |
| Unigene91381 | 3.61 | 3.02 | 4.31 | Glutamate binding protein | Unknown |
| Unigene94127 | 2.85 | 1.4 | 2.2 | Receptor-like kinase | Unknown |
