# Supplementary material for: Transcriptome Analysis of Nicotiana tabacum Infected by Cucumber mosaic virus during Systemic Symptom Development
Source: PLoS One. 2012 Aug 28;7(8):e43447. doi: 10.1371/journal.pone.0043447 (PMC3429483; doi:10.1371/journal.pone.0043447)
Supplement: Table S10 — KEGG-annotated common DEGs at 13 dpi and 16 dpi. (DOC) [file pone.0043447.s015.doc]

Table S10. KEGG-annotated common DEGs at 13 dpi and 16 dpi.

| Gene | Fold change (log_2_R) | | Putative function description | KEGG function class |
| --- | --- | --- | --- | --- |
|  | 13 dpi | 16 dpi |  |  |
| Unigene51575 | 1.88 | 1.79 | Ribose 5-phosphate isomerase A | Metabolism; Carbohydrate Metabolism Metabolism; Energy Metabolism |
| Unigene79750 | 2.8 | 2.12 | Phospholipase C, delta | Metabolism; Carbohydrate Metabolism Environmental Information Processing; Signal Transduction |
| Unigene19450 | 1.53 | 1.9 | UDP-arabinose 4-epimerase | Metabolism; Carbohydrate Metabolism |
| Unigene2493 | 2.8 | 2.36 | Class IV chitinase | Metabolism; Carbohydrate Metabolism |
| Unigene35034 | 1.34 | 1.1 | Chitinase | Metabolism; Carbohydrate Metabolism |
| Unigene41888 | 2.13 | 1.4 | Basic chitinase | Metabolism; Carbohydrate Metabolism |
| Unigene48531 | 1.04 | 1.36 | Pyruvate kinase | Metabolism; Carbohydrate Metabolism |
| Unigene58623 | 3.45 | 1.87 | Endochitinase 3 | Metabolism; Carbohydrate Metabolism |
| Unigene63676 | 2.62 | 4.8 | Basic endochitinase | Metabolism; Carbohydrate Metabolism |
| Unigene71122 | 2.57 | 1.26 | UDP-glucose 4-epimerase [EC:5.1.3.2] | Metabolism; Carbohydrate Metabolism |
| Unigene80198 | 2.11 | 3.51 | Glucan endo-1,3-beta-glucosidase | Metabolism; Carbohydrate Metabolism |
| Unigene83694 | 1.22 | 1.34 | Pectinesterase | Metabolism; Carbohydrate Metabolism |
| Unigene91547 | 2.25 | 1.78 | Pectinesterase | Metabolism; Carbohydrate Metabolism |
| Unigene95361 | -3.89 | -3.15 | Pectinesterase | Metabolism; Carbohydrate Metabolism |
| Unigene19453 | 8.79 | 8.94 | UDP-glucosyl transferase 73C | Metabolism; Glycan Biosynthesis and Metabolism |
| Unigene16993 | 1.61 | 1.5 | Glycine hydroxymethyltransferase | Metabolism; Amino Acid Metabolism Metabolism; Metabolism of Cofactors and Vitamins |
| Unigene38129 | 2.51 | 2.23 | Caffeoyl-coa O-methyltransferase | Metabolism; Amino Acid Metabolism Metabolism; Biosynthesis of Other Secondary Metabolites |
| Unigene86011 | -1.05 | -1.05 | DNA (cytosine-5-)-methyltransferase | Metabolism; Amino Acid Metabolism Genetic Information Processing; Replication and Repair |
| Unigene10873 | 4.7 | 2.09 | Saccharopine dehydrogenase | Metabolism; Amino Acid Metabolism |
| Unigene24728 | 3.39 | 2.94 | 1,2-dihydroxy-3-keto-5-methylthiopentene dioxygenase | Metabolism; Amino Acid Metabolism |
| Unigene25263 | 3.53 | 1.85 | Tryptophan synthase beta chain | Metabolism; Amino Acid Metabolism |
| Unigene85655 | 1.96 | 1.88 | Type 2 proly 4-hydroxylase | Metabolism; Amino Acid Metabolism |
| Unigene85968 | 3.96 | 3.7 | Tryptophan synthase beta chain 2 | Metabolism; Amino Acid Metabolism |
| Unigene93135 | 1.75 | 1.44 | 1-aminocyclopropane-1-carboxylate synthase | Metabolism; Amino Acid Metabolism |
| Unigene85934 | 3.79 | 1.57 | Probable glutathione S-transferase | Metabolism; Metabolism of Other Amino Acid |
| Unigene95363 | 1.71 | 2.89 | Cyp72a55v2 | Metabolism; Lipid Metabolism |
| Unigene21416 | 1.46 | 1.22 | Sphingosine kinase | Metabolism; Lipid Metabolism Environmental Information Processing; Signal Transduction |
| Unigene86611 | 1.63 | 1.4 | Diacylglycerol kinase | Metabolism; Lipid Metabolism Environmental Information Processing; Signal Transduction |
| Unigene84601 | 2.24 | 1.69 | Peroxisomal acyl-coenzyme A oxidase 1 | Metabolism; Lipid Metabolism Cellular Processes; Transport and Catabolism |
| Unigene28251 | 1.84 | 1.96 | Lipoxygenase | Metabolism; Lipid Metabolism |
| Unigene72845 | 1.62 | 1.29 | Serine palmitoyltransferase | Metabolism; Lipid Metabolism |
| Unigene94996 | 1.47 | 1.37 | Lipase-like protein | Metabolism; Lipid Metabolism |
| Unigene6056 | 3.67 | 2.97 | Carbonic anhydrase | Metabolism; Energy Metabolism |
| Unigene8376 | 2.02 | 2.32 | Carbonic anhydrase | Metabolism; Energy Metabolism |
| Unigene8656 | 8.2 | 8.33 | NADH dehydrogenase | Metabolism; Energy Metabolism |
| Unigene92746 | 3.66 | 2.09 | Putative NADH dehydrogenase | Metabolism; Energy Metabolism |
| Unigene10169 | 2.92 | 3.32 | Cytochrome P450-dependent fatty acid hydroxylase | Metabolism; Metabolism of Terpenoids and Polyketides Metabolism; Biosynthesis of Other Secondary Metabolites |
| Unigene22886 | 1.88 | 2.82 | Cytochrome P450 CYP71D47v1 | Metabolism; Metabolism of Terpenoids and Polyketides Metabolism; Biosynthesis of Other Secondary Metabolites |
| Unigene83015 | 3.61 | 2.26 | Elicitor-inducible cytochrome P450 | Metabolism; Metabolism of Terpenoids and Polyketides Metabolism; Biosynthesis of Other Secondary Metabolites |
| Unigene24706 | 2.99 | 1.92 | Cytochrome P450 CYP71B36 | Metabolism; Metabolism of Terpenoids and Polyketides |
| Unigene5173 | 2.15 | 1.85 | Casbene synthase | Metabolism; Metabolism of Terpenoids and Polyketides |
| Unigene593 | 2.02 | 2.26 | Gibberellin 2-oxidase | Metabolism; Metabolism of Terpenoids and Polyketides |
| Unigene64153 | 1.39 | 1.18 | 1,8-cineole synthase | Metabolism; Metabolism of Terpenoids and Polyketides |
| Unigene67261 | 3.96 | 8.67 | Gibberellin 3-beta-dioxygenase | Metabolism; Metabolism of Terpenoids and Polyketides |
| Unigene72141 | 2.13 | 1.15 | Isopentenyl-diphosphate delta-isomerase | Metabolism; Metabolism of Terpenoids and Polyketides |
| Unigene81783 | 9.26 | 9.33 | Cytochrome P450, family 3, subfamily A | Metabolism; Metabolism of Terpenoids and Polyketides |
| Unigene83162 | 2.75 | 2.28 | UDP-glucosyl transferase 73C | Metabolism; Metabolism of Terpenoids and Polyketides |
| Unigene89154 | 3.01 | 1.69 | UDP-glucosyl transferase 73C | Metabolism; Metabolism of Terpenoids and Polyketides |
| Unigene9926 | -1.44 | -1.1 | (+)-Abscisic acid 8'-hydroxylase | Metabolism; Metabolism of Terpenoids and Polyketides |
| Unigene25641 | 2.35 | 1.24 | Type II pantothenate kinase | Metabolism; Metabolism of Cofactors and Vitamins |
| Unigene92101 | 1.45 | 1.74 | Nucleoside-triphosphatase | Metabolism; Metabolism of Cofactors and Vitamins |
| Unigene32838 | 2.7 | 1.48 | Cytochrome P450 CYP92A2v4 | Metabolism; Biosynthesis of Other Secondary Metabolites |
| Unigene5738 | 1.2 | 3.13 | Putative leucoanthocyanidin dioxygenase | Metabolism; Biosynthesis of Other Secondary Metabolites |
| Unigene9643 | 1.97 | 2 | Naringenin 3-dioxygenase | Metabolism; Biosynthesis of Other Secondary Metabolites |
| Unigene2737 | 1.79 | 1.45 | Somatic embryogenesis receptor kinase 1 | Metabolism; Enzyme Families |
| Unigene95339 | 2.08 | 1.21 | Sterile alpha motif and leucine zipper containing kinase AZK | Metabolism; Enzyme Families |
| Unigene46460 | -1.39 | -1.14 | Xyloglucan:xyloglucosyl transferase | Unclassified; Metabolism |
| Unigene14622 | 11.79 | 4.17 | Protein phosphatase | Unclassified; Metabolism |
| Unigene20481 | 3.2 | 2.8 | Ca2+-transporting atpase | Unclassified; Metabolism |
| Unigene2051 | -1.03 | -1.12 | Xyloglucan:xyloglucosyl transferase | Unclassified; Metabolism |
| Unigene4080 | 2.23 | 1.59 | Protein phosphatase | Unclassified; Metabolism |
| Unigene58834 | 2.67 | 2.13 | Xyloglucan:xyloglucosyl transferase | Unclassified; Metabolism |
| Unigene88771 | 2.65 | 2.48 | IAA-amino acid hydrolase | Unclassified; Metabolism |
| Unigene88972 | -1.54 | -1.1 | Xyloglucan:xyloglucosyl transferase | Unclassified; Metabolism |
| Unigene94649 | 3.18 | 1.81 | Protein phosphatase 2C | Unclassified; Metabolism |
| Unigene95031 | 2.39 | 2.43 | Ribonuclease P subunit RPR2 | Genetic Information Processing; Translation |
| Unigene16158 | 10.18 | 9.63 | EREBP (ethylene-responsive element binding protein)-like factor | Genetic Information Processing; Transcription |
| Unigene17386 | 2.48 | 3.45 | Heat shock 70kda protein 1/8 | Genetic Information Processing; Transcription |
| Unigene19341 | 1.19 | 2.49 | Ethylene-responsive transcription factor | Genetic Information Processing; Transcription |
| Unigene23813 | 2.61 | 1.18 | Splicing factor, arginine/serine-rich 2 | Genetic Information Processing; Transcription |
| Unigene3420 | 8.3 | 2.68 | EREBP (ethylene-responsive element binding protein)-like factor | Genetic Information Processing; Transcription |
| Unigene463 | 4.83 | 5.39 | Homeobox-leucine zipper protein | Genetic Information Processing; Transcription |
| Unigene49372 | 3.74 | 9.23 | EREBP (ethylene-responsive element binding protein)-like factor | Genetic Information Processing; Transcription |
| Unigene76761 | 2.8 | 2.76 | Homeobox-leucine zipper protein | Genetic Information Processing; Transcription |
| Unigene81917 | 5.2 | 4.59 | EREBP(ethylene-responsive element binding protein)-like factor | Genetic Information Processing; Transcription |
| Unigene86765 | 1.8 | 1.82 | Integrator complex subunit 11 | Genetic Information Processing; Transcription |
| Unigene87394 | 1.38 | 2.67 | EREBP(ethylene-responsive element binding protein)-like factor | Genetic Information Processing; Transcription |
| Unigene88793 | 1.27 | 2.15 | EREBP(ethylene-responsive element binding protein)-like factor | Genetic Information Processing; Transcription |
| Unigene24544 | -3.38 | -1.53 | Replication factor A1 | Genetic Information Processing; Replication and Repair |
| Unigene14608 | 1.36 | 1.47 | ATP-dependent Clp protease | Genetic Information Processing; Folding, Sorting and Degradation |
| Unigene17020 | 2.85 | 1.77 | Thioredoxin 1 | Genetic Information Processing; Folding, Sorting and Degradation |
| Unigene17433 | -2.56 | -1.03 | Glutaredoxin | Genetic Information Processing; Folding, Sorting and Degradation |
| Unigene25408 | 1.71 | 2.37 | Ubiquitin-protein ligase | Genetic Information Processing; Folding, Sorting and Degradation |
| Unigene30190 | 1.15 | 1.02 | Syntaxin 7 | Genetic Information Processing; Folding, Sorting and Degradation |
| Unigene71745 | 1.64 | 1.24 | E3 ubiquitin-protein ligase | Genetic Information Processing; Folding, Sorting and Degradation |
| Unigene76867 | 1.57 | 1.36 | Protein neuralized | Genetic Information Processing; Folding, Sorting and Degradation |
| Unigene83092 | 1.21 | 1.05 | Ubiquitin-conjugating enzyme E2 I | Genetic Information Processing; Folding, Sorting and Degradation |
| Unigene93995 | 2.61 | 1.86 | Thioredoxin 1 | Genetic Information Processing; Folding, Sorting and Degradation |
| Unigene56426 | 4.5 | 3.09 | Mitochondrial chaperone BCS1 | Unclassified; Genetic Information Processing |
| Unigene7553 | 2.61 | 3.27 | Peptidyl-trna hydrolase, PTH1 family | Unclassified; Genetic Information Processing |
| Unigene24386 | 2.61 | 2.5 | ABC-2 type transport system ATP-binding protein | Environmental Information Processing; Membrane Transport |
| Unigene25242 | 1.71 | 1.05 | ABC transporter B | Environmental Information Processing; Membrane Transport |
| Unigene59546 | -1.39 | -1.07 | ATP-binding cassette, subfamily B (MDR/TAP) | Environmental Information Processing; Membrane Transport |
| Unigene18310 | 9.21 | 8.81 | Aquaporin TIP | Environmental Information Processing; Signaling Molecules and Interaction |
| Unigene24337 | 1.53 | 2.83 | Aquaporin-like protein | Environmental Information Processing; Signaling Molecules and Interaction |
| Unigene43286 | 2.12 | 1.28 | Cysteine proteinase, putative | Environmental Information Processing; Signaling Molecules and Interaction |
| Unigene94597 | 2.16 | 3.02 | Glutamate-gated kainate-type ion channel receptor subunit | Environmental Information Processing; Signaling Molecules and Interaction |
| Unigene24906 | 2.74 | 4.55 | Protein brassinosteroid insensitive 1 | Environmental Information Processing; Signal Transduction |
| Unigene16039 | 3.2 | 4.08 | Serine/threonine protein kinase family protein | Unclassified; Cellular Processes and Signaling |
| Unigene17628 | 2.68 | 2.24 | Putative serine/threonine-protein kinase-like protein | Unclassified; Cellular Processes and Signaling |
| Unigene2074 | 2.07 | 1.5 | Proton-dependent oligopeptide transporter, POT family | Unclassified; Cellular Processes and Signaling |
| Unigene23060 | 2.07 | 2 | Serine/threonine protein kinase family protein | Unclassified; Cellular Processes and Signaling |
| Unigene25450 | 1.61 | 1.77 | S-locus-like receptor protein kinase | Unclassified; Cellular Processes and Signaling |
| Unigene25626 | 2.07 | 1.81 | 5'-AMP-activated protein kinase , catalytic alpha subunit | Unclassified; Cellular Processes and Signaling |
| Unigene26512 | 2.91 | 2.46 | APK2A (protein kinase 2A) | Unclassified; Cellular Processes and Signaling |
| Unigene87012 | 1.87 | 1.74 | Brassinosteroid insensitive 1-associated receptor kinase 1 | Unclassified; Cellular Processes and Signaling |
| Unigene88664 | -1.48 | -1.38 | Brassinosteroid insensitive 1-associated receptor kinase 1 | Unclassified; Cellular Processes and Signaling |
| Unigene89856 | 2.29 | 2.01 | Proton-dependent oligopeptide transporter, POT family | Unclassified; Cellular Processes and Signaling |
| Unigene93327 | 2.45 | 1.47 | Leucine-rich repeat family protein / protein kinase family protein | Unclassified; Cellular Processes and Signaling |
| Unigene93983 | 4.4 | 3.66 | Leucine-rich repeat family protein / protein kinase family protein | Unclassified; Cellular Processes and Signaling |
| Unigene95787 | 1.04 | 1.02 | 2-hydroxyacyl-coa lyase 1 | Cellular Processes; Transport and Catabolism |
| Unigene66869 | 2.1 | 1.43 | Actin | Cellular Processes; Cell Motility |
| Unigene1306 | 2.71 | 2.43 | Interleukin-1 receptor-associated kinase 4 | Cellular Processes; Cell Growth and Death |
| Unigene15522 | 2.32 | 1.97 | Interleukin-1 receptor-associated kinase 4 | Cellular Processes; Cell Growth and Death |
| Unigene4157 | 1.49 | 1.71 | Interleukin-1 receptor-associated kinase 4 | Cellular Processes; Cell Growth and Death |
| Unigene83363 | 2.18 | 1.75 | Interleukin-1 receptor-associated kinase 4 | Cellular Processes; Cell Growth and Death |
| Unigene95065 | 2.11 | 2.34 | Interleukin-1 receptor-associated kinase 4 | Cellular Processes; Cell Growth and Death |
| Unigene17666 | 2.51 | 1.54 | Calcium-binding protein CML | Organismal Systems; Environmental Adaptation |
| Unigene12163 | 1.78 | 1.76 | Disease resistance protein RPM1 | Organismal Systems; Environmental Adaptation |
| Unigene47774 | 2.38 | 1.23 | Cyclic nucleotide gated channel | Organismal Systems; Environmental Adaptation |
| Unigene66034 | 1.53 | 2.13 | WRKY transcription factor 33 | Organismal Systems; Environmental Adaptation |
| Unigene70534 | 2.7 | 1.96 | RIN4, RPM1 interacting protein 4 | Organismal Systems; Environmental Adaptation |
| Unigene76965 | 1.38 | 1.65 | Chitin elicitor receptor kinase 1 | Organismal Systems; Environmental Adaptation |
| Unigene78166 | 3.55 | 2.16 | Transcription factor MYC2 | Organismal Systems; Environmental Adaptation |
| Unigene78975 | 2.44 | 2.47 | Calcium-dependent protein kinase | Organismal Systems; Environmental Adaptation |
| Unigene84545 | 1.95 | 2.16 | Calcium-binding protein CML | Organismal Systems; Environmental Adaptation |
| Unigene89415 | 1.84 | 1.7 | Serine/threonine-protein kinase PBS1 | Organismal Systems; Environmental Adaptation |
| Unigene89561 | 1.68 | 1.94 | Mitogen-activated protein kinase kinase kinase 1, plant | Organismal Systems; Environmental Adaptation |
| Unigene93151 | 1.49 | 1.71 | LRR receptor-like serine/threonine-protein kinase FLS2 | Organismal Systems; Environmental Adaptation |
| Unigene94280 | 2.36 | 3.86 | Serine/threonine-protein kinase PBS1 | Organismal Systems; Environmental Adaptation |
| Unigene94936 | 2.28 | 1.04 | Mitogen-activated protein kinase kinase kinase 1, plant | Organismal Systems; Environmental Adaptation |
| Unigene95370 | 1.47 | 1.82 | Disease resistance protein RPM1 | Organismal Systems; Environmental Adaptation |
| Unigene90404 | 1.41 | 1.7 | Exocyst complex component 7 | Unknown |
| Unigene25039 | 9.82 | 10.91 | Chloroplast nucleoid DNA binding protein | Unknown |
| Unigene51812 | 3.12 | 2.03 | Putative hydrolase of the HAD superfamily | Unknown |
| Unigene59157 | 1.61 | 1.02 | Hypothetical protein | Unknown |
| Unigene9176 | 2.09 | 1.82 | Hypothetical protein | Unknown |
