# Supplementary material for: Transcriptome Analysis of Nicotiana tabacum Infected by Cucumber mosaic virus during Systemic Symptom Development
Source: PLoS One. 2012 Aug 28;7(8):e43447. doi: 10.1371/journal.pone.0043447 (PMC3429483; doi:10.1371/journal.pone.0043447)
Supplement: Table S11 — KEGG-annotated common DEGs at 20 dpi and 16 dpi. (DOC) [file pone.0043447.s016.doc]

Table S11. KEGG-annotated common DEGs at 20 dpi and 16 dpi.

| Gene | Fold change (log_2_R) | | Putative function description | KEGG function class |
| --- | --- | --- | --- | --- |
|  | 13 dpi | 16 dpi |  |  |
| Unigene51575 | 1.79 | 2.61 | Ribose 5-phosphate isomerase A | Metabolism; Carbohydrate Metabolism Metabolism; Energy Metabolism |
| Unigene79750 | 2.12 | 1.95 | Phospholipase C, delta | Metabolism; Carbohydrate Metabolism Environmental Information Processing; Signal Transduction |
| Unigene19450 | 1.9 | 1.89 | UDP-arabinose 4-epimerase | Metabolism; Carbohydrate Metabolism |
| Unigene21818 | 2.14 | 2.08 | Beta-amylase | Metabolism; Carbohydrate Metabolism |
| Unigene41888 | 1.4 | 1.33 | Basic chitinase | Metabolism; Carbohydrate Metabolism |
| Unigene48531 | 1.36 | 1.2 | Pyruvate kinase | Metabolism; Carbohydrate Metabolism |
| Unigene58623 | 1.87 | 1.33 | Endochitinase 3 | Metabolism; Carbohydrate Metabolism |
| Unigene63676 | 4.8 | 4.84 | Basic endochitinase | Metabolism; Carbohydrate Metabolism |
| Unigene80198 | 3.51 | 1.93 | Glucan endo-1,3-beta-glucosidase | Metabolism; Carbohydrate Metabolism |
| Unigene91547 | 1.78 | 1.79 | Pectinesterase | Metabolism; Carbohydrate Metabolism |
| Unigene11449 | 1.46 | 2.36 | Beta-galactosidase | Metabolism; Carbohydrate Metabolism Metabolism; Glycan Biosynthesis and Metabolism |
| Unigene16993 | 1.5 | 2.96 | Glycine hydroxymethyltransferase | Metabolism; Amino Acid Metabolism Metabolism; Metabolism of Cofactors and Vitamins |
| Unigene68284 | 1.18 | 2 | S-adenosylmethionine synthetase | Metabolism; Amino Acid metabolism Metabolism; Biosynthesis of plant hormones |
| Unigene38129 | 2.23 | 1.46 | Caffeoyl-coa O-methyltransferase | Metabolism; Amino Acid Metabolism Metabolism; Biosynthesis of Other Secondary Metabolites |
| Unigene10873 | 2.09 | 1.69 | Saccharopine dehydrogenase | Metabolism; Amino Acid Metabolism |
| Unigene24728 | 2.94 | 2.11 | 1,2-dihydroxy-3-keto-5-methylthiopentene dioxygenase | Metabolism; Amino Acid Metabolism |
| Unigene25263 | 1.85 | 4.23 | Tryptophan synthase beta chain | Metabolism; Amino Acid Metabolism |
| Unigene85655 | 1.88 | 2.89 | Type 2 proly 4-hydroxylase | Metabolism; Amino Acid Metabolism |
| Unigene85968 | 3.7 | 3.7 | Tryptophan synthase beta chain 2 | Metabolism; Amino Acid Metabolism |
| Unigene54832 | 1.31 | 1.31 | Glutathione S-transferase | Metabolism; Metabolism of Other Amino Acids |
| Unigene85934 | 1.57 | 2.22 | Probable glutathione S-transferase | Metabolism; Metabolism of Other Amino Acids |
| Unigene84601 | 1.69 | 1.22 | Peroxisomal acyl-coenzyme A oxidase 1 | Metabolism; Lipid Metabolism Cellular Processes; Transport and Catabolism |
| Unigene2972 | 1.18 | 2.02 | Hydroperoxide dehydratase | Metabolism; Lipid Metabolism |
| Unigene94996 | 1.37 | 3.38 | Lipase-like protein | Metabolism; Lipid Metabolism |
| Unigene95168 | 9.51 | 3.34 | Nucleoside-triphosphatase | Metabolism; Nucleotide Metabolism Metabolism; Metabolism of Cofactors and Vitamins |
| Unigene13668 | 1.15 | -1.87 | V-type H+-transporting atpase subunit F | Metabolism; Energy Metabolism |
| Unigene8376 | 2.32 | 4.04 | Carbonic anhydrase | Metabolism; Energy Metabolism |
| Unigene92746 | 2.09 | 1.8 | Putative NADH dehydrogenase | Metabolism; Energy Metabolism |
| Unigene64249 | 2.31 | 3.22 | 1-deoxy-D-xylulose-5 -phosphate synthase | Metabolism; Metabolism of Terpenoids and Polyketides Metabolism; Biosynthesis of plant hormones |
| Unigene22886 | 2.82 | 3.95 | Cytochrome P450 CYP71D47v1 | Metabolism; Metabolism of Terpenoids and Polyketides Metabolism; Biosynthesis of Other Secondary Metabolites |
| Unigene83015 | 2.26 | 2.41 | Elicitor-inducible cytochrome P450 | Metabolism; Metabolism of Terpenoids and Polyketides Metabolism; Biosynthesis of Other Secondary Metabolites |
| Unigene11396 | 2.62 | 4.13 | Xanthoxin dehydrogenase | Metabolism; Metabolism of Terpenoids and Polyketides |
| Unigene64153 | 1.18 | 1.73 | 1,8-cineole synthase | Metabolism; Metabolism of Terpenoids and Polyketides |
| Unigene67261 | 8.67 | 8.67 | Gibberellin 3-beta-dioxygenase | Metabolism; Metabolism of Terpenoids and Polyketides |
| Unigene72141 | 1.15 | 1.9 | Isopentenyl-diphosphate delta-isomerase | Metabolism; Metabolism of Terpenoids and Polyketides |
| Unigene81783 | 9.33 | 8.52 | Cytochrome P450, family 3, subfamily A | Metabolism; Metabolism of Terpenoids and Polyketides |
| Unigene83162 | 2.28 | 2.58 | UDP-glucosyl transferase 73C | Metabolism; Metabolism of Terpenoids and Polyketides |
| Unigene89154 | 1.69 | 2.14 | UDP-glucosyl transferase 73C | Metabolism; Metabolism of Terpenoids and Polyketides |
| Unigene9926 | -1.1 | -2.92 | (+)-Abscisic acid 8'-hydroxylase | Metabolism; Metabolism of Terpenoids and Polyketides |
| Unigene83884 | 1.94 | 1.43 | 4-coumarate--coa ligase | Metabolism; Metabolism of Cofactors and Vitamins Metabolism; Biosynthesis of Other Secondary Metabolites |
| Unigene25641 | 1.24 | 1.57 | Type II pantothenate kinase | Metabolism; Metabolism of Cofactors and Vitamins |
| Unigene92101 | 1.74 | 3.6 | Nucleoside-triphosphatase | Metabolism; Metabolism of Cofactors and Vitamins |
| Unigene16795 | -1.37 | -1.87 | 2-oxoglutarate-dependent dioxygenase | Metabolism; Biosynthesis of Other Secondary Metabolites |
| Unigene24929 | 1.44 | -2.03 | Cyanohydrin beta-glucosyltransferase | Metabolism; Biosynthesis of Other Secondary Metabolites |
| Unigene32838 | 1.48 | 2.35 | Cytochrome P450 CYP92A2v4 | Metabolism; Biosynthesis of Other Secondary Metabolites |
| Unigene5738 | 3.13 | 3.13 | Putative leucoanthocyanidin dioxygenase | Metabolism; Biosynthesis of Other Secondary Metabolites |
| Unigene46460 | -1.14 | -1.25 | Xyloglucan:xyloglucosyl transferase | Unclassified; Metabolism |
| Unigene12720 | 9.81 | 8.74 | Cysteine proteinase, putative | Unclassified; Metabolism |
| Unigene14622 | 4.17 | 2.1 | Protein phosphatase | Unclassified; Metabolism |
| Unigene88771 | 2.48 | 2.1 | IAA-amino acid hydrolase | Unclassified; Metabolism |
| Unigene88972 | -1.1 | -1.05 | Xyloglucan:xyloglucosyl transferase | Unclassified; Metabolism |
| Unigene94649 | 1.81 | 1.3 | Protein phosphatase 2C | Unclassified; Metabolism |
| Unigene3214 | -2.19 | -1.85 | Translation initiation factor IF-1 | Genetic Information Processing; Translation |
| Unigene71408 | 1.1 | -1.81 | Protein phosphatase 2 (formerly 2A), regulatory subunit A | Genetic Information Processing; Translation |
| Unigene95031 | 2.43 | 1.57 | Ribonuclease P subunit RPR2 | Genetic Information Processing; Translation |
| Unigene10799 | -1.23 | -1.41 | Homeobox-leucine zipper protein | Genetic Information Processing; Transcription |
| Unigene19341 | 2.49 | 5.14 | Ethylene-responsive transcription factor | Genetic Information Processing; Transcription |
| Unigene23813 | 1.18 | 2.35 | Pre-mrna-splicing factor | Genetic Information Processing; Transcription |
| Unigene3420 | 2.68 | 2.82 | EREBP (ethylene-responsive element binding protein)-like factor | Genetic Information Processing; Transcription |
| Unigene34270 | 2.44 | -1.93 | EREBP (ethylene-responsive element binding protein)-like factor | Genetic Information Processing; Transcription |
| Unigene76761 | 2.76 | 2.29 | Homeobox-leucine zipper protein | Genetic Information Processing; Transcription |
| Unigene81917 | 4.59 | 5.32 | EREBP(ethylene-responsive element binding protein)-like factor | Genetic Information Processing; Transcription |
| Unigene24544 | -1.53 | -1.7 | Replication factor A1 | Genetic Information Processing; Replication and Repair |
| Unigene66976 | 1.58 | -1.04 | Ubiquitin-conjugating enzyme E2 variant | Genetic Information Processing; Replication and Repair |
| Unigene14608 | 1.47 | 2.43 | ATP-dependent Clp protease | Genetic Information Processing; Folding, Sorting and Degradation |
| Unigene17020 | 1.77 | 1.2 | Thioredoxin 1 | Genetic Information Processing; Folding, Sorting and Degradation |
| Unigene17433 | -1.03 | -3.06 | Glutaredoxin | Genetic Information Processing; Folding, Sorting and Degradation |
| Unigene25408 | 2.37 | 3.52 | Ubiquitin-protein ligase | Genetic Information Processing; Folding, Sorting and Degradation |
| Unigene71745 | 1.24 | 2.02 | E3 ubiquitin-protein ligase | Genetic Information Processing; Folding, Sorting and Degradation |
| Unigene76867 | 1.36 | 1.35 | Protein neuralized | Genetic Information Processing; Folding, Sorting and Degradation |
| Unigene80693 | 2.08 | 2.57 | Chaperonin groel | Genetic Information Processing; Folding, Sorting and Degradation |
| Unigene88098 | 1.07 | -1.24 | Ubiquitin-activating enzyme E1 | Genetic Information Processing; Folding, Sorting and Degradation |
| Unigene93143 | 1.81 | 2.13 | Syntaxin 7 | Genetic Information Processing; Folding, Sorting and Degradation |
| Unigene93995 | 1.86 | 1.58 | Thioredoxin 1 | Genetic Information Processing; Folding, Sorting and Degradation |
| Unigene94077 | 8.59 | 8.74 | ATP-dependent Clp protease ATP-binding subunit clpc | Genetic Information Processing; Folding, Sorting and Degradation |
| Unigene18310 | 8.81 | 8.99 | Aquaporin TIP | Environmental Information Processing; Signaling Molecules and Interaction |
| Unigene24337 | 2.83 | 2.67 | Aquaporin-like protein | Environmental Information Processing; Signaling Molecules and Interaction |
| Unigene87846 | 2.8 | 2.97 | Glutamate receptor, ionotropic, other eukaryote | Environmental Information Processing; Signaling Molecules and Interaction |
| Unigene94597 | 3.02 | 2.53 | Glutamate-gated kainate-type ion channel receptor subunit | Environmental Information Processing; Signaling Molecules and Interaction |
| Unigene24906 | 4.55 | 9.7 | Protein brassinosteroid insensitive 1 | Environmental Information Processing; Signal Transduction |
| Unigene16039 | 4.08 | 1.24 | Serine/threonine protein kinase family protein | Unclassified; Cellular Processes and Signaling |
| Unigene17628 | 2.24 | 2.86 | Putative serine/threonine-protein kinase-like protein | Unclassified; Cellular Processes and Signaling |
| Unigene25450 | 1.77 | 1.59 | S-locus-like receptor protein kinase | Unclassified; Cellular Processes and Signaling |
| Unigene73482 | 1.82 | -2.22 | Brassinosteroid insensitive 1-associated receptor kinase 1 | Unclassified; Cellular Processes and Signaling |
| Unigene87012 | 1.74 | 3.93 | Brassinosteroid insensitive 1-associated receptor kinase 1 | Unclassified; Cellular Processes and Signaling |
| Unigene88292 | 8.74 | 8.15 | Leucine-rich repeat family protein  / protein kinase family protein | Unclassified; Cellular Processes and Signaling |
| Unigene89856 | 2.01 | 2.57 | Proton-dependent oligopeptide transporter, POT family | Unclassified; Cellular Processes and Signaling |
| Unigene93983 | 3.66 | 3.51 | Leucine-rich repeat family protein / protein kinase family protein | Unclassified; Cellular Processes and Signaling |
| Unigene4157 | 1.71 | 1.37 | Interleukin-1 receptor-associated kinase 4 | Cellular Processes; Cell Growth and Death |
| Unigene92648 | 9.59 | 1.86 | Interleukin-1 receptor-associated kinase 4 | Cellular Processes; Cell Growth and Death |
| Unigene95757 | 2.21 | 2.76 | Interleukin-1 receptor-associated kinase 4 | Cellular Processes; Cell Growth and Death |
| Unigene47774 | 1.23 | 2.05 | Cyclic nucleotide gated channel | Organismal Systems; Environmental Adaptation |
| Unigene66034 | 2.13 | 2.84 | WRKY transcription factor 33 | Organismal Systems; Environmental Adaptation |
| Unigene70534 | 1.96 | 1.84 | RIN4, RPM1 interacting protein 4 | Organismal Systems; Environmental Adaptation |
| Unigene78166 | 2.16 | 2.21 | Transcription factor MYC2 | Organismal Systems; Environmental Adaptation |
| Unigene84545 | 2.16 | 1.21 | Calcium-binding protein CML | Organismal Systems; Environmental Adaptation |
| Unigene89415 | 1.7 | 1.06 | Serine/threonine-protein kinase PBS1 | Organismal Systems; Environmental Adaptation |
| Unigene94280 | 3.86 | 2.15 | Serine/threonine-protein kinase PBS1 | Organismal Systems; Environmental Adaptation |
| Unigene16738 | -1.09 | 1.11 | Predicted protein | Unknown |
| Unigene25039 | 10.91 | 8.15 | Chloroplast nucleoid DNA binding protein | Unknown |
| Unigene51812 | 2.03 | 2.34 | Putative hydrolase of the HAD superfamily | Unknown |
| Unigene90404 | 1.7 | 1.71 | Exocyst complex component 7 | Unknown |
