# Supplementary material for: Transcriptome Analysis of Nicotiana tabacum Infected by Cucumber mosaic virus during Systemic Symptom Development
Source: PLoS One. 2012 Aug 28;7(8):e43447. doi: 10.1371/journal.pone.0043447 (PMC3429483; doi:10.1371/journal.pone.0043447)
Supplement: Table S12 — KEGG-annotated common DEGs at 20 dpi and 6 dpi. (DOC) [file pone.0043447.s017.doc]

Table S12. KEGG-annotated common DEGs at 20 dpi and 6 dpi.

| Gene | Fold change (log_2_R) | | Putative function description | KEGG function class |
| --- | --- | --- | --- | --- |
|  | 13 dpi | 16 dpi |  |  |
| Unigene95560 | -1.67 | -1.11 | Ribose-phosphate pyrophosphokinase | Metabolism; Carbohydrate Metabolism Metabolism; Nucleotide Metabolism |
| Unigene74525 | -1.96 | -2.60 | Formyltetrahydrofolate deformylase | Metabolism; Carbohydrate Metabolism Metabolism; Metabolism of Cofactors and Vitamins |
| Unigene68373 | 2.12 | 2.51 | S-(hydroxymethyl)glutathione dehydrogenase / alcohol dehydrogenase | Metabolism; Carbohydrate Metabolism Metabolism; Amino Acid Metabolism Metabolism; Lipid Metabolism Metabolism; Xenobiotics Biodegradation and Metabolism |
| Unigene6331 | -1.20 | -1.71 | Phosphoenolpyruvate carboxylase [EC:4.1.1.31] | Metabolism; Carbohydrate Metabolism Metabolism; Energy Metabolism |
| Unigene93704 | 1.75 | 2.16 | Fructose-bisphosphate aldolase, class I | Metabolism; Carbohydrate Metabolism Metabolism; Energy Metabolism |
| Unigene94789 | 2.54 | 2.10 | Aldehyde dehydrogenase (NAD+) | Metabolism; Carbohydrate Metabolism Metabolism; Amino Acid Metabolism Metabolism; Lipid Metabolism Metabolism; Metabolism of Terpenoids and Polyketides |
| Unigene21806 | 1.84 | 1.02 | Beta-galactosidase [EC:3.2.1.23] | Metabolism; Carbohydrate Metabolism Metabolism; Glycan Biosynthesis and Metabolism |
| Unigene10843 | 1.47 | 1.99 | Pectate lyase | Metabolism; Carbohydrate Metabolism |
| Unigene19431 | -1.92 | -2.32 | UDP-glucuronate 4-epimerase | Metabolism; Carbohydrate Metabolism |
| Unigene19450 | 1.89 | 2.54 | UDP-arabinose 4-epimerase | Metabolism; Carbohydrate Metabolism |
| Unigene20944 | -1.68 | -3.52 | Pectinesterase | Metabolism; Carbohydrate Metabolism |
| Unigene230 | 1.50 | 1.75 | 1-aminocyclopropane-1-carboxylate deaminase | Metabolism; Carbohydrate Metabolism |
| Unigene24303 | 1.09 | 1.68 | Glucose-1-phosphate adenylyltransferase | Metabolism; Carbohydrate Metabolism |
| Unigene25728 | -2.23 | -3.38 | Glucan endo-1,3-beta-D-glucosidase | Metabolism; Carbohydrate Metabolism |
| Unigene31932 | -1.90 | -2.34 | Malate dehydrogenase | Metabolism; Carbohydrate Metabolism |
| Unigene3219 | 3.74 | 2.99 | Stachyose synthetase | Metabolism; Carbohydrate Metabolism |
| Unigene41888 | 1.33 | 3.41 | Basic chitinase | Metabolism; Carbohydrate Metabolism |
| Unigene42166 | -2.36 | -2.25 | Isoflavone reductase | Metabolism; Carbohydrate Metabolism |
| Unigene48531 | 1.20 | 1.39 | Pyruvate kinase | Metabolism; Carbohydrate Metabolism |
| Unigene48602 | -2.14 | -2.67 | UDP-glucuronate 4-epimerase | Metabolism; Carbohydrate Metabolism |
| Unigene57190 | -1.23 | -1.39 | Starch phosphorylase | Metabolism; Carbohydrate Metabolism |
| Unigene58623 | 1.33 | 1.51 | Endochitinase 3 | Metabolism; Carbohydrate Metabolism |
| Unigene63676 | 4.84 | 8.02 | Basic endochitinase | Metabolism; Carbohydrate Metabolism |
| Unigene75792 | 1.07 | 1.19 | Fructose-bisphosphate aldolase, class I | Metabolism; Carbohydrate Metabolism |
| Unigene80198 | 1.93 | 3.48 | Glucan endo-1,3-beta-glucosidase | Metabolism; Carbohydrate Metabolism |
| Unigene85505 | 1.13 | 1.18 | 3-hydroxyisobutyryl-coa hydrolase | Metabolism; Carbohydrate Metabolism |
| Unigene86275 | -1.13 | -1.02 | 3-isopropylmalate/(R)-2-methylmalate dehydratase large subunit | Metabolism; Carbohydrate Metabolism |
| Unigene91547 | 1.79 | 1.61 | Pectinesterase | Metabolism; Carbohydrate Metabolism |
| Unigene92932 | -1.41 | -1.67 | L-ascorbate oxidase | Metabolism; Carbohydrate Metabolism |
| Unigene93913 | -1.17 | -1.38 | Starch synthase | Metabolism; Carbohydrate Metabolism |
| Unigene94446 | -1.14 | -1.71 | Endoglucanase | Metabolism; Carbohydrate Metabolism |
| Unigene9482 | -2.02 | -3.21 | UDP-glucuronate 4-epimerase [EC:5.1.3.6] | Metabolism; Carbohydrate Metabolism |
| Unigene95416 | -1.55 | -2.13 | Starch synthase | Metabolism; Carbohydrate Metabolism |
| Unigene95672 | -1.71 | -1.70 | Starch phosphorylase | Metabolism; Carbohydrate Metabolism |
| Unigene11449 | 2.36 | 1.28 | Beta-galactosidase | Metabolism; Carbohydrate Metabolism Metabolism; Glycan Biosynthesis and Metabolism |
| Unigene86959 | -1.67 | -1.32 | Ribulose-bisphosphate carboxylase large chain | Metabolism; Carbohydrate Metabolism Metabolism; Energy Metabolism |
| Unigene95011 | -1.80 | -1.99 | Ribulose-bisphosphate carboxylase large chain [EC:4.1.1.39] | Metabolism; Carbohydrate Metabolism Metabolism; Energy Metabolism |
| Unigene93448 | -1.31 | -1.43 | 1-phosphatidylinositol-4-phosphate 5-kinase | Metabolism; Carbohydrate Metabolism Environmental Information Processing; Signal Transduction Cellular Processes; Transport and Catabolism |
| Unigene84547 | -1.74 | -2.21 | Cellulose synthase A | Metabolism; Glycan Biosynthesis and Metabolism |
| Unigene13692 | -1.58 | -1.94 | Anthocyanin 5-O-glucosyltransferase | Metabolism; Glycan Biosynthesis and Metabolism Metabolism; Biosynthesis of Other Secondary Metabolites |
| Unigene16177 | -4.40 | -2.65 | Heparan-alpha-glucosaminide N-acetyltransferase | Metabolism; Glycan Biosynthesis and Metabolism |
| Unigene88786 | 2.15 | 1.91 | Lysosomal alpha-mannosidase | Metabolism; Glycan Biosynthesis and Metabolism |
| Unigene91753 | -1.73 | -2.19 | Peptide-O-fucosyltransferase | Metabolism; Glycan Biosynthesis and Metabolism |
| Unigene9438 | 1.26 | 1.09 | Callose synthase | Metabolism; Glycan Biosynthesis and Metabolism |
| Unigene74757 | -2.42 | -1.88 | Glutamate-1-semialdehyde 2,1-aminomutase [EC:5.4.3.8] | Metabolism; Amino Acid Metabolism Metabolism; Metabolism of Cofactors and Vitamins |
| Unigene90916 | -2.36 | -1.68 | Aminomethyltransferase | Metabolism; Amino Acid Metabolism Metabolism; Energy Metabolism Metabolism; Metabolism of Cofactors and Vitamins |
| Unigene24090 | -2.58 | -1.97 | Serine O-acetyltransferase | Metabolism; Amino Acid Metabolism Metabolism; Energy Metabolism |
| Unigene26529 | -1.54 | -1.42 | Cystathionine gamma-synthase | Metabolism; Amino Acid Metabolism Metabolism; Energy Metabolism |
| Unigene61912 | -1.74 | -1.06 | Sulfotransferase | Metabolism; Amino Acid Metabolism Metabolism; Biosynthesis of Other Secondary Metabolites |
| Unigene10873 | 1.69 | 2.81 | Saccharopine dehydrogenase | Metabolism; Amino Acid Metabolism |
| Unigene15130 | 1.20 | 1.44 | Dihydrodipicolinate synthase | Metabolism; Amino Acid Metabolism |
| Unigene18588 | 9.34 | 4.06 | Asparagine synthetase | Metabolism; Amino Acid Metabolism |
| Unigene24061 | -2.46 | -2.52 | Aminotransferase | Metabolism; Amino Acid Metabolism |
| Unigene24728 | 2.11 | 2.19 | 1,2-dihydroxy-3-keto-5-methylthiopentene dioxygenase | Metabolism; Amino Acid Metabolism |
| Unigene5488 | -1.99 | -1.94 | Phosphoserine phosphatase | Metabolism; Amino Acid Metabolism |
| Unigene62631 | -1.40 | -1.76 | 3-dehydroquinate dehydratase / shikimate dehydrogenase [EC:4.2.1.10 1.1.1.25] | Metabolism; Amino Acid Metabolism |
| Unigene65276 | 8.15 | 3.77 | Phenylalanine ammonia-lyase | Metabolism; Amino Acid Metabolism |
| Unigene70561 | 2.84 | 1.27 | Aspartate aminotransferase | Metabolism; Amino Acid Metabolism |
| Unigene85655 | 2.89 | 2.65 | Type 2 proly 4-hydroxylase | Metabolism; Amino Acid Metabolism |
| Unigene85968 | 3.70 | 1.66 | Tryptophan synthase beta chain 2 | Metabolism; Amino Acid Metabolism |
| Unigene88170 | -2.31 | -2.14 | Histone-lysine N-methyltransferase SETD2 | Metabolism; Amino Acid Metabolism |
| Unigene92974 | -1.19 | -1.61 | 1,2-dihydroxy-3-keto-5-methylthiopentene dioxygenase | Metabolism; Amino Acid Metabolism |
| Unigene7149 | 1.71 | 2.49 | Methionyl-trna synthetase | Metabolism; Amino Acid Metabolism Genetic Information Processing; Translation |
| Unigene95235 | 1.39 | 2.73 | Urease | Metabolism; Amino Acid Metabolism Metabolism; Nucleotide Metabolism |
| Unigene21196 | -2.07 | -2.26 | Peroxidase | Metabolism; Amino Acid Metabolism Metabolism; Energy Metabolism |
| Unigene59232 | 1.61 | 2.04 | Serine palmitoyltransferase | Metabolism; Amino Acid Metabolism Metabolism; Lipid Metabolism |
| Unigene12603 | -1.94 | -1.42 | Cyanohydrin beta-glucosyltransferase | Metabolism; Metabolism of Other Amino Acids Metabolism; Glycan Biosynthesis and Metabolism |
| Unigene54832 | 1.31 | 2.88 | Glutathione S-transferase | Metabolism; Metabolism of Other Amino Acids |
| Unigene73945 | -1.66 | -1.49 | Beta-cyano-L-alanine hydratase/nitrilase | Metabolism; Metabolism of Other Amino Acids |
| Unigene85934 | 2.22 | 2.34 | Probable glutathione S-transferase | Metabolism; Metabolism of Other Amino Acids |
| Unigene84601 | 1.22 | 1.16 | Peroxisomal acyl-coenzyme A oxidase 1 | Metabolism; Lipid Metabolism Cellular Processes; Transport and Catabolism |
| Unigene18684 | 1.78 | 1.85 | 3-oxo-5-alpha-steroid 4-dehydrogenase 1 | Metabolism; Lipid Metabolism |
| Unigene50262 | -1.59 | -2.81 | Acyl-[acyl-carrier-protein] desaturase | Metabolism; Lipid Metabolism |
| Unigene57543 | -1.21 | -2.14 | [acyl-carrier-protein] S-malonyltransferase | Metabolism; Lipid Metabolism |
| Unigene61266 | -1.43 | -1.02 | Lathosterol oxidase | Metabolism; Lipid Metabolism |
| Unigene65529 | -1.73 | -2.43 | Omega-6 fatty acid desaturase (delta-12 desaturase) | Metabolism; Lipid Metabolism |
| Unigene7972 | 1.45 | 1.50 | Palmitoyl-protein thioesterase | Metabolism; Lipid Metabolism |
| Unigene94996 | 3.38 | 2.16 | Lipase-like protein | Metabolism; Lipid Metabolism |
| Unigene55614 | -1.26 | -1.14 | DNA-directed RNA polymerase II subunit G | Metabolism; Nucleotide Metabolism Genetic Information Processing; Transcription |
| Unigene17499 | 1.59 | 1.69 | Adenylate kinase | Metabolism; Nucleotide Metabolism |
| Unigene51572 | -1.46 | -1.44 | Carbonic anhydrase | Metabolism; Energy Metabolism |
| Unigene5556 | -2.16 | -1.47 | NADH dehydrogenase (ubiquinone) 1 alpha/beta subcomplex 1 | Metabolism; Energy Metabolism |
| Unigene833 | 1.35 | 1.27 | Cytochrome c oxidase subunit Vb | Metabolism; Energy Metabolism |
| Unigene8376 | 4.04 | 3.33 | Carbonic anhydrase | Metabolism; Energy Metabolism |
| Unigene85798 | 1.32 | 1.15 | Cytochrome c oxidase subunit Vb [EC:1.9.3.1] | Metabolism; Energy Metabolism |
| Unigene87698 | 2.16 | 2.51 | NADH dehydrogenase (ubiquinone) 1 alpha subcomplex 6 | Metabolism; Energy Metabolism |
| Unigene87733 | -2.07 | -2.16 | Phosphoribulokinase | Metabolism; Energy Metabolism |
| Unigene90547 | -1.39 | -1.33 | Photosystem II oxygen-evolving enhancer protein | Metabolism; Energy Metabolism |
| Unigene91984 | -3.16 | -1.85 | Nitrate reductase (NADH) | Metabolism; Energy Metabolism |
| Unigene92746 | 1.80 | 2.94 | Putative NADH dehydrogenase | Metabolism; Energy Metabolism |
| Unigene94289 | 1.07 | 1.53 | V-type H+-transporting atpase 21kda proteolipid subunit | Metabolism; Energy Metabolism |
| Unigene95188 | -2.14 | -2.21 | Photosystem II psba protein | Metabolism; Energy Metabolism |
| Unigene95377 | -1.82 | -2.29 | Photosystem II psbc protein | Metabolism; Energy Metabolism |
| Unigene2842 | -1.10 | -1.62 | Cytochrome P450 | Metabolism; Metabolism of Terpenoids and Polyketides Metabolism; Biosynthesis of Other Secondary Metabolites |
| Unigene69985 | -3.65 | -2.90 | Cytochrome P450 | Metabolism; Metabolism of Terpenoids and Polyketides Metabolism; Biosynthesis of Other Secondary Metabolites |
| Unigene79304 | -2.15 | -1.99 | Cytochrome P450 CYP77A4 | Metabolism; Metabolism of Terpenoids and Polyketides Metabolism; Biosynthesis of Other Secondary Metabolites |
| Unigene64249 | 3.22 | 1.92 | 1-deoxy-D-xylulose-5 -phosphate synthase | Metabolism; Metabolism of Terpenoids and Polyketides Metabolism; Biosynthesis of plant hormones |
| Unigene22886 | 3.95 | 3.17 | Cytochrome P450 CYP71D47v1 | Metabolism; Metabolism of Terpenoids and Polyketides Metabolism; Biosynthesis of Other Secondary Metabolites |
| Unigene83015 | 2.41 | 5.13 | Elicitor-inducible cytochrome P450 | Metabolism; Metabolism of Terpenoids and Polyketides Metabolism; Biosynthesis of Other Secondary Metabolites |
| Unigene11396 | 4.13 | 2.48 | Xanthoxin dehydrogenase | Metabolism; Metabolism of Terpenoids and Polyketides |
| Unigene2829 | -2.57 | -3.65 | Xanthoxin dehydrogenase | Metabolism; Metabolism of Terpenoids and Polyketides |
| Unigene33294 | -3.09 | -2.63 | Xanthoxin dehydrogenase | Metabolism; Metabolism of Terpenoids and Polyketides |
| Unigene35240 | -2.07 | -2.41 | UDP-glucosyl transferase | Metabolism; Metabolism of Terpenoids and Polyketides |
| Unigene67261 | 8.67 | 8.44 | Gibberellin 3-beta-dioxygenase | Metabolism; Metabolism of Terpenoids and Polyketides |
| Unigene72141 | 1.90 | 2.09 | Isopentenyl-diphosphate delta-isomerase | Metabolism; Metabolism of Terpenoids and Polyketides |
| Unigene75469 | -1.99 | -1.83 | Cytokinin dehydrogenase | Metabolism; Metabolism of Terpenoids and Polyketides |
| Unigene81783 | 8.52 | 8.52 | Cytochrome P450, family 3, subfamily A | Metabolism; Metabolism of Terpenoids and Polyketides |
| Unigene83162 | 2.58 | 1.52 | UDP-glucosyl transferase 73C | Metabolism; Metabolism of Terpenoids and Polyketides |
| Unigene84140 | -1.71 | -1.18 | Zeaxanthin epoxidase | Metabolism; Metabolism of Terpenoids and Polyketides |
| Unigene87689 | 11.05 | 9.88 | Casbene synthase | Metabolism; Metabolism of Terpenoids and Polyketides |
| Unigene89154 | 2.14 | 2.58 | UDP-glucosyl transferase 73C | Metabolism; Metabolism of Terpenoids and Polyketides |
| Unigene93691 | -1.28 | -1.53 | Carotenoid isomerase | Metabolism; Metabolism of Terpenoids and Polyketides |
| Unigene9926 | -2.92 | -2.42 | (+)-Abscisic acid 8'-hydroxylase | Metabolism; Metabolism of Terpenoids and Polyketides |
| Unigene86807 | 1.75 | 1.64 | Dehydrogenase/reductase SDR family member 4 | Metabolism; Metabolism of Cofactors and Vitamins Cellular Processes; Transport and Catabolism |
| Unigene83884 | 1.43 | 2.06 | 4-coumarate--coa ligase | Metabolism; Metabolism of Cofactors and Vitamins Metabolism; Biosynthesis of Other Secondary Metabolites |
| Unigene23591 | -2.15 | -4.05 | Nucleoside-triphosphatase | Metabolism; Metabolism of Cofactors and Vitamins |
| Unigene25124 | -1.44 | -1.35 | 8-amino-7-oxononanoate synthase | Metabolism; Metabolism of Cofactors and Vitamins |
| Unigene32315 | -2.57 | -2.40 | Uroporphyrinogen decarboxylase | Metabolism; Metabolism of Cofactors and Vitamins |
| Unigene40160 | -1.12 | -2.76 | Protochlorophyllide reductase | Metabolism; Metabolism of Cofactors and Vitamins |
| Unigene45545 | -1.87 | -1.87 | Glutamine amidotransferase | Metabolism; Metabolism of Cofactors and Vitamins |
| Unigene49818 | -8.42 | -2.97 | Protochlorophyllide reductase | Metabolism; Metabolism of Cofactors and Vitamins |
| Unigene5396 | -2.50 | -2.94 | Chlorophyllase | Metabolism; Metabolism of Cofactors and Vitamins |
| Unigene58898 | -1.32 | -1.35 | Imilar to Acylamino-acid-releasing enzyme | Metabolism; Metabolism of Cofactors and Vitamins |
| Unigene66747 | -1.60 | -1.53 | Porphobilinogen synthase | Metabolism; Metabolism of Cofactors and Vitamins |
| Unigene69414 | -3.14 | -2.55 | Homogentisate solanesyltransferase | Metabolism; Metabolism of Cofactors and Vitamins |
| Unigene89919 | -1.00 | -1.02 | Magnesium chelatase subunit I | Metabolism; Metabolism of Cofactors and Vitamins |
| Unigene16795 | -1.87 | -1.51 | 2-oxoglutarate-dependent dioxygenase | Metabolism; Biosynthesis of Other Secondary Metabolites |
| Unigene25148 | -1.53 | 10.50 | 2-oxoglutarate-dependent dioxygenase | Metabolism; Biosynthesis of Other Secondary Metabolites |
| Unigene32838 | 2.35 | 3.95 | Cytochrome P450 CYP92A2v4 | Metabolism; Biosynthesis of Other Secondary Metabolites |
| Unigene5738 | 3.13 | 3.71 | Putative leucoanthocyanidin dioxygenase | Metabolism; Biosynthesis of Other Secondary Metabolites |
| Unigene67323 | 3.62 | 2.66 | Cinnamyl-alcohol dehydrogenase | Metabolism; Biosynthesis of Other Secondary Metabolites |
| Unigene75969 | -2.02 | -1.15 | Anthocyanin 5-O-glucosyltransferase | Metabolism; Biosynthesis of Other Secondary Metabolites |
| Unigene93347 | 1.33 | 2.71 | Cinnamoyl-coa reductase | Metabolism; Biosynthesis of Other Secondary Metabolites |
| Unigene10704 | 1.39 | 3.31 | Serine/threonine kinase 16 | Metabolism; Enzyme Families |
| Unigene12950 | -1.38 | -1.67 | Cell division protease ftsh | Metabolism; Enzyme Families |
| Unigene24744 | 2.11 | 1.91 | Ubiquitin carboxyl-terminal hydrolase 5/13 | Metabolism; Enzyme Families |
| Unigene35083 | -2.39 | -3.05 | Phytepsin | Metabolism; Enzyme Families |
| Unigene58598 | -2.94 | -2.07 | Cathepsin A | Metabolism; Enzyme Families |
| Unigene81807 | 1.70 | 1.40 | Serine/threonine-protein kinase/endoribonuclease IRE1 | Metabolism; Enzyme Families |
| Unigene83690 | 2.50 | 1.81 | Sterile alpha motif and leucine zipper containing kinase AZK | Metabolism; Enzyme Families |
| Unigene83748 | -1.98 | -1.90 | Serine/threonine-protein kinase/endoribonuclease IRE1 | Metabolism; Enzyme Families |
| Unigene92695 | 1.05 | 1.32 | Mitochondrial processing peptidase | Metabolism; Enzyme Families |
| Unigene93155 | -2.75 | -3.14 | Ubiquitin carboxyl-terminal hydrolase 16/45 | Metabolism; Enzyme Families |
| Unigene16513 | 1.19 | 1.75 | Phospholipid-translocating atpase | Unclassified; Metabolism |
| Unigene12720 | 8.74 | 13.61 | Cysteine proteinase, putative | Unclassified; Metabolism |
| Unigene14651 | -1.75 | -2.75 | Dual specificity protein phosphatase | Unclassified; Metabolism |
| Unigene25090 | -1.64 | -1.77 | [ribulose-bisphosphate carboxylase]-lysine N-methyltransferase | Unclassified; Metabolism |
| Unigene71609 | -2.73 | -1.72 | Alternative oxidase | Unclassified; Metabolism |
| Unigene83846 | -1.17 | -1.66 | 4a-hydroxytetrahydrobiopterin dehydratase | Unclassified; Metabolism |
| Unigene89377 | 1.27 | 1.25 | Protein phosphatase [EC:3.1.3.16] | Unclassified; Metabolism |
| Unigene90643 | -1.78 | -2.36 | [ribulose-bisphosphate carboxylase]-lysine N-methyltransferase | Unclassified; Metabolism |
| Unigene94270 | -2.78 | -8.97 | Xyloglucan:xyloglucosyl transferase | Unclassified; Metabolism |
| Unigene94649 | 1.30 | 1.54 | Protein phosphatase 2C | Unclassified; Metabolism |
| Unigene1193 | -1.43 | -1.45 | Methionyl-trna synthetase | Genetic Information Processing; Translation |
| Unigene24946 | 1.90 | 3.17 | Large subunit ribosomal protein l10e | Genetic Information Processing; Translation |
| Unigene62858 | -1.63 | -1.34 | Large subunit ribosomal protein L2 | Genetic Information Processing; Translation |
| Unigene71316 | -1.48 | -1.05 | Large subunit ribosomal protein L15 | Genetic Information Processing; Translation |
| Unigene77874 | 1.15 | 1.16 | Large subunit ribosomal protein l10e | Genetic Information Processing; Translation |
| Unigene78002 | -1.64 | -1.10 | Large subunit ribosomal protein l35e | Genetic Information Processing; Translation |
| Unigene79890 | -1.22 | -1.03 | Asparaginyl-trna synthetase | Genetic Information Processing; Translation |
| Unigene86427 | 2.26 | 1.88 | Large subunit ribosomal protein l7e | Genetic Information Processing; Translation |
| Unigene87085 | 1.50 | 1.28 | Translation initiation factor eif-1A | Genetic Information Processing; Translation |
| Unigene88400 | -2.19 | -1.85 | Elongation factor EF-G | Genetic Information Processing; Translation |
| Unigene91576 | -1.01 | -1.35 | Large subunit ribosomal protein l22e | Genetic Information Processing; Translation |
| Unigene92892 | 1.16 | 1.22 | Large subunit ribosomal protein l3e | Genetic Information Processing; Translation |
| Unigene95031 | 1.57 | 1.86 | Ribonuclease P subunit RPR2 | Genetic Information Processing; Translation |
| Unigene95833 | -1.29 | -1.87 | Small subunit ribosomal protein S5 | Genetic Information Processing; Translation |
| Unigene95882 | 1.47 | 1.96 | Protein phosphatase 2 (formerly 2A), regulatory subunit B' | Genetic Information Processing; Translation |
| Unigene10799 | -1.41 | -1.99 | Homeobox-leucine zipper protein | Genetic Information Processing; Transcription |
| Unigene14958 | -2.51 | -1.46 | Myb proto-oncogene protein, plant | Genetic Information Processing; Transcription |
| Unigene18502 | 1.12 | 1.47 | U4/U6.U5 tri-snrnp component SNU23 | Genetic Information Processing; Transcription |
| Unigene19341 | 5.14 | 2.68 | Ethylene-responsive transcription factor | Genetic Information Processing; Transcription |
| Unigene19394 | 1.04 | 1.17 | Plasminogen activator inhibitor 1 RNA-binding protein | Genetic Information Processing; Transcription |
| Unigene21233 | -1.23 | -1.40 | MADS-box transcription factor | Genetic Information Processing; Transcription |
| Unigene23505 | -1.88 | -1.48 | ATP-dependent RNA helicase | Genetic Information Processing; Transcription |
| Unigene23813 | 2.35 | 2.22 | Pre-mrna-splicing factor | Genetic Information Processing; Transcription |
| Unigene50147 | 8.25 | 2.94 | RAV-like factor | Genetic Information Processing; Transcription |
| Unigene58721 | -1.22 | -1.02 | Far upstream element-binding protein | Genetic Information Processing; Transcription |
| Unigene64449 | -1.96 | -1.38 | EREBP (ethylene-responsive element binding protein)-like factor | Genetic Information Processing; Transcription |
| Unigene71588 | 2.36 | 2.80 | Homeobox protein cut-like | Genetic Information Processing; Transcription |
| Unigene72797 | -1.58 | -1.52 | Splicing factor 3B subunit 5 | Genetic Information Processing; Transcription |
| Unigene76218 | -1.25 | -1.05 | RNA polymerase primary sigma factor | Genetic Information Processing; Transcription |
| Unigene86613 | -2.09 | -2.24 | EREBP (ethylene-responsive element binding protein)-like factor | Genetic Information Processing; Transcription |
| Unigene86913 | 2.58 | 1.73 | Splicing factor, arginine/serine-rich 1/9 | Genetic Information Processing; Transcription |
| Unigene91833 | -1.19 | 1.08 | U4/U6 small nuclear ribonucleoprotein PRP4 | Genetic Information Processing; Transcription |
| Unigene94191 | -1.19 | -1.49 | Prp8 binding protein | Genetic Information Processing; Transcription |
| Unigene94312 | -1.56 | -1.51 | Coiled-coil domain-containing protein 130 | Genetic Information Processing; Transcription |
| Unigene10786 | -3.13 | -2.65 | DNA-3-methyladenine glycosylase II | Genetic Information Processing; Replication and Repair |
| Unigene24544 | -1.70 | -2.44 | Replication factor A1 | Genetic Information Processing; Replication and Repair |
| Unigene37405 | 1.71 | 2.19 | Ubiquitin-conjugating enzyme E2 variant | Genetic Information Processing; Replication and Repair |
| Unigene6391 | 2.19 | 2.43 | Protein HIRA/HIR1 | Genetic Information Processing; Replication and Repair |
| Unigene65305 | -1.74 | -2.66 | Replication factor A1 | Genetic Information Processing; Replication and Repair |
| Unigene80741 | -1.01 | -1.08 | Proliferating cell nuclear antigen | Genetic Information Processing; Replication and Repair |
| Unigene82940 | -1.98 | -1.22 | DNA topoisomerase I | Genetic Information Processing; Replication and Repair |
| Unigene86724 | -1.61 | -1.34 | Structural maintenance of chromosome 1 | Genetic Information Processing; Replication and Repair |
| Unigene92855 | -2.08 | -1.52 | Histone demethylase JARID1 | Genetic Information Processing; Replication and Repair |
| Unigene17433 | -3.06 | -2.00 | Glutaredoxin | Genetic Information Processing; Folding, Sorting and Degradation |
| Unigene19644 | 2.64 | 2.46 | ATP-dependent RNA helicase DDX6/DHH1 | Genetic Information Processing; Folding, Sorting and Degradation |
| Unigene24023 | -1.68 | -2.38 | Exosome complex component RRP46 | Genetic Information Processing; Folding, Sorting and Degradation |
| Unigene25408 | 3.52 | 7.67 | Ubiquitin-protein ligase | Genetic Information Processing; Folding, Sorting and Degradation |
| Unigene2973 | 1.25 | 1.89 | E3 ubiquitin-protein ligase RNF115/126 | Genetic Information Processing; Folding, Sorting and Degradation |
| Unigene62479 | -2.47 | -2.78 | Chaperonin groel | Genetic Information Processing; Folding, Sorting and Degradation |
| Unigene70095 | 1.21 | 1.80 | Translocon-associated protein subunit alpha | Genetic Information Processing; Folding, Sorting and Degradation |
| Unigene71745 | 2.02 | 3.03 | E3 ubiquitin-protein ligase | Genetic Information Processing; Folding, Sorting and Degradation |
| Unigene76164 | 1.94 | 2.61 | SKP1-like protein 3 | Genetic Information Processing; Folding, Sorting and Degradation |
| Unigene76867 | 1.35 | 1.34 | Protein neuralized | Genetic Information Processing; Folding, Sorting and Degradation |
| Unigene84192 | 1.50 | 1.51 | Ubiquitin-conjugating enzyme E2 I | Genetic Information Processing; Folding, Sorting and Degradation |
| Unigene87058 | 2.21 | 2.19 | Cereblon | Genetic Information Processing; Folding, Sorting and Degradation |
| Unigene89366 | 1.46 | 2.48 | E3 ubiquitin-protein ligase RNF14 | Genetic Information Processing; Folding, Sorting and Degradation |
| Unigene89879 | 2.28 | 2.26 | S-phase kinase-associated protein 1 | Genetic Information Processing; Folding, Sorting and Degradation |
| Unigene93027 | 1.45 | 1.42 | Hsp70-interacting protein | Genetic Information Processing; Folding, Sorting and Degradation |
| Unigene93143 | 2.13 | 2.10 | Syntaxin 7 | Genetic Information Processing; Folding, Sorting and Degradation |
| Unigene94123 | 1.82 | 2.30 | Vesicle-associated membrane protein 72 | Genetic Information Processing; Folding, Sorting and Degradation |
| Unigene95302 | -1.80 | -2.07 | T-complex protein 1 subunit theta | Genetic Information Processing; Folding, Sorting and Degradation |
| Unigene9742 | -2.09 | -1.20 | Transitional endoplasmic reticulum atpase | Genetic Information Processing; Folding, Sorting and Degradation |
| Unigene61630 | -2.04 | -1.76 | Peptidyl-trna hydrolase, PTH2 family | Unclassified; Genetic Information Processing |
| Unigene88312 | -2.08 | -1.46 | Peptidylprolyl isomerase [EC:5.2.1.8] | Unclassified; Genetic Information Processing |
| Unigene17275 | -1.61 | -1.70 | MFS transporter, PHS family, inorganic phosphate transporter | Environmental Information Processing; Membrane Transport |
| Unigene74600 | 1.53 | 1.82 | ATP-binding cassette, subfamily B (MDR/TAP), member 1 | Environmental Information Processing; Membrane Transport |
| Unigene24337 | 2.67 | 1.73 | Aquaporin-like protein | Environmental Information Processing; Signaling Molecules and Interaction |
| Unigene49747 | 2.41 | 2.53 | Rab family | Environmental Information Processing; Signaling Molecules and Interaction |
| Unigene87671 | -1.43 | -1.28 | Rab family, other | Environmental Information Processing; Signaling Molecules and Interaction |
| Unigene87846 | 2.97 | 2.46 | Glutamate receptor, ionotropic, other eukaryote | Environmental Information Processing; Signaling Molecules and Interaction |
| Unigene87933 | 1.45 | 1.03 | Ras-related protein Rab-8A | Environmental Information Processing; Signaling Molecules and Interaction |
| Unigene93721 | -1.23 | -1.75 | ADP-ribosylation factor-like 1 | Environmental Information Processing; Signaling Molecules and Interaction |
| Unigene9409 | 1.44 | 1.22 | Rab family, other | Environmental Information Processing; Signaling Molecules and Interaction |
| Unigene94597 | 2.53 | 1.40 | Glutamate-gated kainate-type ion channel receptor subunit | Environmental Information Processing; Signaling Molecules and Interaction |
| Unigene18857 | 1.89 | 1.83 | Transducin (beta)-like 1 | Environmental Information Processing; Signal Transduction |
| Unigene23527 | -2.70 | -2.58 | Solute carrier family 25 (mitochondrial carrier; adenine nucleotide translocator) | Environmental Information Processing; Signal Transduction |
| Unigene24906 | 9.70 | 13.62 | Protein brassinosteroid insensitive 1 | Environmental Information Processing; Signal Transduction |
| Unigene25453 | -1.71 | -2.75 | Protein brassinosteroid insensitive 1 | Environmental Information Processing; Signal Transduction |
| Unigene87665 | 2.18 | 2.62 | Probable serine/threonine-protein kinase drka | Environmental Information Processing; Signal Transduction |
| Unigene82714 | -1.07 | 1.89 | Calmodulin | Environmental Information Processing; Signal Transduction Organismal Systems; Environmental Adaptation |
| Unigene16039 | 1.24 | 5.22 | Serine/threonine protein kinase family protein | Unclassified; Cellular Processes and Signaling |
| Unigene17628 | 2.86 | 3.21 | Putative serine/threonine-protein kinase-like protein | Unclassified; Cellular Processes and Signaling |
| Unigene25450 | 1.59 | 3.02 | S-locus-like receptor protein kinase | Unclassified; Cellular Processes and Signaling |
| Unigene33586 | -2.58 | -1.23 | LRR receptor-like serine/threonine-protein kinase FLS2 | Unclassified; Cellular Processes and Signaling |
| Unigene73482 | -2.22 | 1.34 | Brassinosteroid insensitive 1-associated receptor kinase 1 | Unclassified; Cellular Processes and Signaling |
| Unigene82895 | 1.67 | 1.24 | Protein-tyrosine phosphatase | Unclassified; Cellular Processes and Signaling |
| Unigene87012 | 3.93 | 3.41 | Brassinosteroid insensitive 1-associated receptor kinase 1 | Unclassified; Cellular Processes and Signaling |
| Unigene88007 | 1.06 | 2.01 | Bola protein | Unclassified; Cellular Processes and Signaling |
| Unigene88292 | 8.15 | 8.94 | Leucine-rich repeat family protein  / protein kinase family protein | Unclassified; Cellular Processes and Signaling |
| Unigene94475 | 1.58 | 1.63 | K00924|1|3e-33|138|ath:AT1G30270| [EC:2.7.1.-] | Unclassified; Cellular Processes and Signaling |
| Unigene43964 | -1.24 | -2.32 | Tubulin alpha | Cellular Processes; Transport and Catabolism |
| Unigene5940 | 1.32 | 2.60 | Peroxisomal 2,4-dienoyl-coa reductase | Cellular Processes; Transport and Catabolism |
| Unigene63592 | -3.51 | -1.97 | Autophagy-related protein 12 | Cellular Processes; Transport and Catabolism |
| Unigene6483 | -3.01 | -1.61 | Stromal membrane-associated protein | Cellular Processes; Transport and Catabolism |
| Unigene83890 | 1.48 | 1.35 | ADP-ribosylation factor gtpase-activating protein 1 | Cellular Processes; Transport and Catabolism |
| Unigene87541 | -1.16 | -1.28 | Charged multivesicular body protein 2B | Cellular Processes; Transport and Catabolism |
| Unigene93767 | -1.34 | -1.57 | Natural resistance-associated macrophage protein | Cellular Processes; Transport and Catabolism |
| Unigene1715 | -2.03 | -4.24 | Maintenance of ploidy protein MOB1 (MPS1 binder 1) | Cellular Processes; Cell Growth and Death |
| Unigene73034 | -1.10 | -1.97 | Interleukin-1 receptor-associated kinase 4 | Cellular Processes; Cell Growth and Death |
| Unigene92648 | 1.86 | 4.15 | Interleukin-1 receptor-associated kinase 4 | Cellular Processes; Cell Growth and Death |
| Unigene94512 | -1.12 | -1.57 | Cyclin-dependent kinase regulatory subunit CKS1 | Cellular Processes; Cell Growth and Death |
| Unigene95757 | 2.76 | 3.72 | Interleukin-1 receptor-associated kinase 4 | Cellular Processes; Cell Growth and Death |
| Unigene17446 | 1.26 | 1.27 | WRKY transcription factor 33 | Organismal Systems; Environmental Adaptation |
| Unigene39638 | 4.05 | 11.99 | WRKY transcription factor 33 | Organismal Systems; Environmental Adaptation |
| Unigene47774 | 2.05 | 1.68 | Cyclic nucleotide gated channel | Organismal Systems; Environmental Adaptation |
| Unigene66034 | 2.84 | 8.01 | WRKY transcription factor 33 | Organismal Systems; Environmental Adaptation |
| Unigene66226 | -1.39 | -1.60 | Heat shock protein 90kda beta | Organismal Systems; Environmental Adaptation |
| Unigene70534 | 1.84 | 2.72 | RIN4, RPM1 interacting protein 4 | Organismal Systems; Environmental Adaptation |
| Unigene84545 | 1.21 | 3.29 | Calcium-binding protein CML | Organismal Systems; Environmental Adaptation |
| Unigene88277 | -1.48 | -1.75 | Serine/threonine-protein kinase WNK1 [EC:2.7.11.1] | Organismal Systems; Environmental Adaptation |
| Unigene89415 | 1.06 | 1.59 | Serine/threonine-protein kinase PBS1 | Organismal Systems; Environmental Adaptation |
| Unigene93108 | -2.15 | -4.69 | Transcription factor MYC2 | Organismal Systems; Environmental Adaptation |
| Unigene94280 | 2.15 | 4.46 | Serine/threonine-protein kinase PBS1 | Organismal Systems; Environmental Adaptation |
| Unigene90404 | 1.71 | 2.93 | Exocyst complex component 7 | Unknown |
| Unigene16738 | 1.11 | 1.27 | Predicted protein | Unknown |
| Unigene17757 | 2.13 | 1.52 | Predicted protein | Unknown |
| Unigene18694 | 1.89 | 2.15 | Insulysin-like | Unknown |
| Unigene2442 | 1.59 | 1.18 | Hypothetical protein | Unknown |
| Unigene25039 | 8.15 | 12.69 | Chloroplast nucleoid DNA binding protein | Unknown |
| Unigene25091 | -1.97 | -1.79 | Hypothetical protein | Unknown |
| Unigene25206 | 3.62 | 3.68 | SNF4, homolog of yeast sucrose nonfermenting 4 | Unknown |
| Unigene74634 | 2.57 | 1.07 | SNF1-related kinases | Unknown |
| Unigene82 | 1.44 | 2.21 | Putative hydrolase of the HAD superfamily | Unknown |
| Unigene86202 | -1.24 | -1.26 | Hypothetical protein | Unknown |
| Unigene89630 | -1.06 | -1.53 | SULA; binding / catalytic/ coenzyme binding | Unknown |
| Unigene91381 | 9.70 | 3.61 | Glutamate binding protein | Unknown |
